# Sleep and nighttime behavior disorders in older adults: associations with hypercholesterolemia and hypertriglyceridemia at baseline, and a predictive analysis of incident cases at 12 months follow-up

**DOI:** 10.1186/s12944-024-02302-x

**Published:** 2024-09-28

**Authors:** Asma Hallab, for the Alzheimer’s Disease Neuroimaging Initiative

**Affiliations:** 1 Biologie Intégrative et Physiologie – Parcours Neurosciences Cellulaires et Integrées, Faculté des Sciences et Ingénierie, Campus Pierre Et Marie Curie, Sorbonne Université, Paris, France; 2 Pathologies du Sommeil, Faculté de Médecine, Hopital Universitaire Pitié-Salpêtrière. Sorbonne Université, Paris, France; 3 Charité Universitätsmedizin – Berlin, Corporate Member of Freie Universität Berlin and Humboldt-Universität Zu Berlin, Charitéplatz 1, Berlin, 10117 Germany

**Keywords:** Sleep, Aging, Dyslipidemia, Triglyceride, Cholesterol, BMI

## Abstract

**Introduction:**

Sleep disorders, particularly insomnia and obstructive sleep apnea, are associated with dyslipidemia in the general population. The study’s aim was to explore the association between pathological Cholesterol and Triglyceride levels, and sleep and nighttime behavior disorders (SNBD) in older adults, whether they might predict SNBD onset, and to emphasize the role of body mass index (BMI) in this association.

**Methods:**

Alzheimer’s Disease Neuroimaging Initiative (ADNI) population with complete Cholesterol, Triglyceride, SNBD, and neurocognitive data were included. Logistic regression was performed to study the association between hypercholesterolemia, hypertriglyceridemia, and SNBD at baseline and at 12 months. Relevant confounders, particularly BMI, were adjusted for.

**Results:**

Among the 2,216 included cases, 1,045 (47%) were females, and the median age was 73 years (IQR: 68, 78). At baseline, 357 (16%) had SNBD and 327 (18%) at 12 months; 187 of them were incident cases.

There were more cases of baseline SNBD in the hypertriglyceridemia group than in those without (19% vs. 14%, *P*-value = 0.003). Similarly, more follow-up SNBD cases had hypertriglyceridemia at baseline (21% vs. 16%, *P*-value = 0.025). SNBD cases at baseline had significantly higher serum Triglyceride levels than those without (132 vs. 118mg/dL, *P*-value < 0.001).

Only hypertriglyceridemia was significantly associated with baseline SNBD (crude OR = 1.43, 95%*CI*: 1.13,1.80, *P*-value = 0.003), even after adjustment for confounding factors (adj. OR = 1.36, 95%*CI*: 1.06,1.74, *P*-value = 0.016) and (BMI-adj. OR = 1.29, 95%*CI*: 1.00,1.66, *P-*value = 0.048). None of the dyslipidemia forms did predict incident cases at 12 months.

**Conclusions:**

Hypertriglyceridemia, but not hypercholesterolemia, was associated with higher odds of SNBD. The association was independent of BMI. None of the dyslipidemia forms did predict incident SNBD over 12 months. Sleep disorders should motivate a systematic screening of dyslipidemia in older adults and vice versa.

**Supplementary Information:**

The online version contains supplementary material available at 10.1186/s12944-024-02302-x.

## Introduction

Sleep disorders represent a large spectrum of symptoms defining an alteration of sleep quality, structure, chronobiology, duration, and associated breathing and movement disorders [1]. Insomnia and obstructive sleep apnea (OSA) have a high prevalence worldwide; both are associated with cardiovascular and neuropsychiatric risk factors and affected persons are exposed to higher morbidity and mortality rates [2–5]. The incidence of sleep disorders increases with age. In addition to the physiological decrease in sleep hours during the aging process; neurodegeneration, neuroendocrine, and sleep disorders define a more complex bidirectional association [6]. Old patients with sleep disorders, particularly insomnia, have higher risks of cognitive decline, and those with cognitive impairment are more susceptible to progressing into dementia [7–9]. Moreover, older patients with neurocognitive disorders are at higher risk of experiencing neurodegeneration-related sleep behavior and movement disorders [10].

The relationship between sleep disorders and metabolic and cardiovascular pathologies is largely reported in the literature [11, 12]. Defined as the association between diabetes, dyslipidemia, hypertonia, and visceral adiposity, the metabolic syndrome is a well-established risk factor for cardiovascular disorders and is related to higher morbidity and mortality rates [13]. Most published studies evaluated sleep disorders quantitatively based on sleep duration or qualitatively depending on the subjective perception of sleep quality [14–17]. Studies on sleep disorders objectified by the study partner of older patients and their association with dyslipidemia are rare. Moreover, there is limited data on whether dyslipidemia might predict prospectively sleep disorders. It is also unclear how much this association depends on body mass index (BMI), particularly in older adults. The overall study question was whether hypercholesterolemia or hypertriglyceridemia, independent of BMI, are associated with sleep and nighttime behavior disorders (SNBD) in advanced age groups.

The aims of this study were (1) to explore the association between dyslipidemia and informant-perceived SNBD in older adults at baseline, and (2) to evaluate whether dyslipidemia at baseline might predict incident cases of SNBD over 12 months of follow-up.

## Methods

This manuscript has been prepared and reported according to STROBE guidelines [18].

### Study population

The studied population is part of the Alzheimer’s Disease Neuroimaging Initiative (ADNI) cohort, from which only cases with complete data required for the current analysis were included. Dr Michael W. Weiner is ADNI's principal investigator. ADNI is a non-interventional longitudinal study. Study participants are older adults recruited at 59 centers around the United States and Canada, who underwent an observational follow-up and where biological, genetic, neuroimaging, and neuropsychiatric information was assessed at several time points. Participants from different phases (ADNI 1, go, 2, and 3) were eligible for the current analysis. The study was performed according to the Declaration of Helsinki and ethical approval was obtained from the Internal Reviewing Board corresponding to each participating site. Written consent was obtained from all ADNI study participants. Data, ethical approval, enrollment, and protocols can be found at https://adni.loni.usc.edu.

### Cholesterol and Triglyceride measurements

Serum Cholesterol and Triglyceride levels were assessed at baseline and mainly reported in mg/dL. Laboratory normal ranges were 0—199 mg/dL for Cholesterol and 0—149 mg/dL for Triglyceride. Defect and duplicated measurements were checked for each individual and removed based on the date and time of the reported result. Serum levels corresponding to 200 mg/dL for Cholesterol and 150 mg/dL for Triglyceride are largely recognized clinical cutoff values for hypercholesterolemia and hypertriglyceridemia, respectively [19, 20]. Owing to the larger use of mg/dL as a unit worldwide, values in mmol/L were converted to mg/dL:

$$\mathrm{Cholesterol}\;(\mathrm{mg}/\mathrm{dL})\;=\;\mathrm{Cholesterol}\;(\mathrm{mmol}/\mathrm L)\;\times\;38.67$$

$$\mathrm{Triglycerides}\;(\mathrm{mg}/\mathrm{dL})\;=\;\mathrm{Triglycerides}\;(\mathrm{mmol}/\mathrm L)\;\times\;88.57$$

### Sleep and nighttime behavior disorders

The assessment of SNBD was based on the neuropsychiatric inventory questionnaire (NPI/NPI-Q) filled by study partners of included participants [21, 22]. The item related to sleep disorders in NPI/NPI-Q covered the following questions:

“Does the patient have difficulty sleeping (do not count as present if the patient simply gets up once or twice per night only to go to the bathroom and falls back asleep immediately)? Is he/she up at night? Does he/she wander at night, get dressed, or disturb your sleep?”

If this question was answered with yes, details on the following questions were then collected:

1. “Does the patient have difficulty falling asleep?”.
2. “Does the patient get up during the night?”.
3. “Does the patient wander, pace, or get involved in inappropriate activities at night?”.
4. “Does the patient awaken you during the night?”.
5. “Does the patient wake up at night, dress, and plan to go out, thinking that it is morning and time to start the day?”.
6. “Does the patient awaken too early in the morning, earlier than was his/her habit?”.
7. “Does the patient sleep excessively during the day?”.
8. “Does the patient have any other nighttime behaviors that bother you and we haven’t talked about?”.

### Cognitive tests

Cognition was assessed based on the Alzheimer’s Disease Assessment Score with 13 items (ADAS_13_), Mini-Mental Status Examination (MMSE) total score, Clinical Dementia Rating (CDR) total score, CDR-sum of boxes (CDR-SB), and Functional Activities Questionnaire (FAQ) total score. Moreover, depression symptoms were reported based on the total Geriatric Depression Scale (GDS) score. People with severe depression were initially excluded from ADNI and therefore included participants are either non- or mildly depressed. The included cases were either healthy controls (HC), participants with mild cognitive impairment (MCI), or dementia.

### Body-mass index

Weight and Height data were checked to ensure the plausibility of the values and units. Weight at baseline was considered and converted to “Kilogram” when the unit of measurement was “Pounds”:

$$\mathrm{Kilograms}=\;2.20462\;\ast\;\mathrm{Pounds}$$

Similarly, height was converted to “Meters” when the unit of measurement was “Inches”:

$$\mathrm{Meters}=\;0.0254\;\ast\;\mathrm{Inches}$$

BMI was calculated based on the formula weight (Kg) /Height (m) ^2^ [23].

### Inclusion criteria

**Fig. 1:**
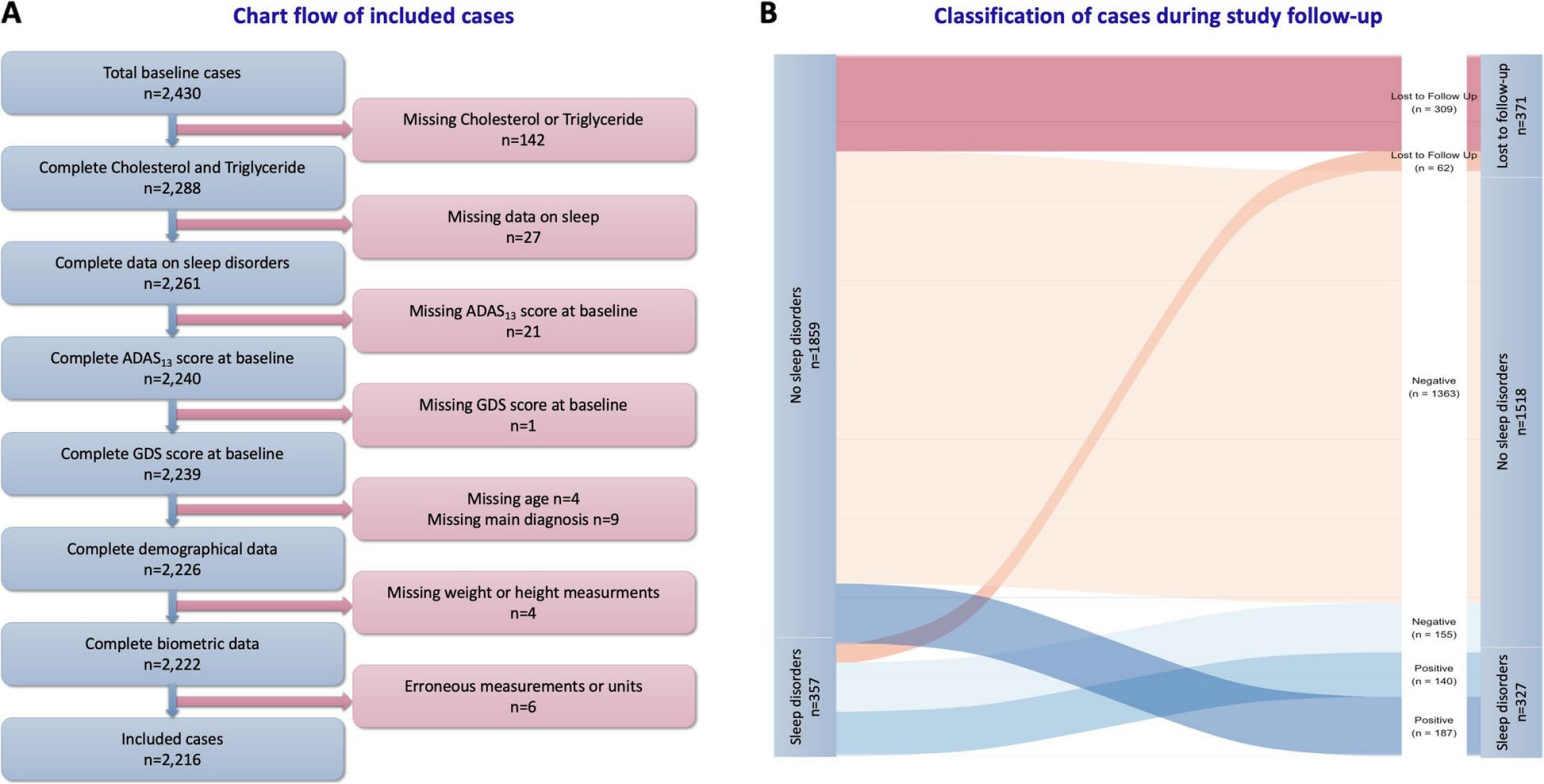
Characteristics of the study population. **A** Chart flow of included studies. **B** Classification of cases during study follow-up

After excluding 142 participants without Cholesterol or Triglyceride measurements, 27 without complete NPI/NPI-Q, 21 missing total ADAS_13_ and one missing GDS score at baseline, nine missing baseline main diagnosis, and four missing complete demographic data (age), four missing weight or height at baseline and six have erroneous measurements or units, a total of 2,216 study participants were included in the analysis (Fig. 1A). Among those 371 were lost to follow-up at 12 months (Fig. 1B).

### Statistical analysis

The statistical analysis was performed by RStudio version 2024–04. Continuous data was reported as median (Inter-quartile range (IQR)) and count data as number (percentage (%)). Kruskal–Wallis rank sum test and Pearson’s Chi-squared test were performed to compare groups and for each analysis, the *P*-value was reported. Spearman correlation between serum Cholesterol, Triglyceride, age, and scores of cognitive tests, was performed and correlation coefficients were reported. The association between SNBD and dyslipidemia at baseline was evaluated using logistic regression with SNBD as a dependent binary variable, and dyslipidemia as an independent binary variable. Models were adjusted for age, sex, racial profile, educational level, GDS total score, Apolipoprotein (APOE) ε4 status, main diagnosis related to cognitive status, and BMI, as follows:

- Model 1: crude logistic regression analysis,
- Model 2: adjusted for age, sex, racial profile, educational level, cognition-related main diagnosis, GDS total score, APOE ε4 status,
- Model 3: model 2 + BMI.

Incident cases of SNBD were calculated as new positive cases at 12 months of follow-up, amongst cases that were negative at baseline. Prediction analysis was based on logistic regression with incident SNBD at 12 months follow-up as a dependent binary variable, and dyslipidemia at baseline as an independent binary variable. The same confounding factors were adjusted for. For each model odds ratio (OR), 95% confidence interval (*CI*), and *P*-value were reported. The statistical significance level was set at 0.05.

## Results

### Characteristics of the study population

Among the 2,216 included cases, 1,045 (47%) were females, and 1,171 (53%) were males. The median age was 73 (IQR: 68, 78), and 786 (%) were HC, 1,060 (%) had MCI, and 370 (%) were diagnosed with dementia. The difference in median age between groups was statistically significant (72, 73, and 75 years, respectively, *P*-value < 0.001).

At baseline, 357 (16%) study participants had sleep and nighttime behavior disorders, according to their study partner. At 12 months of follow-up, 327 (18%) participants had positive sleep disorder scores, among which 187 were incident cases (Fig. 1B).

The median ADAS_13_ total score was 14 (IQR: 9, 22), the median GDS total score was 1.0 (IQR: 0, 2.0), and the median FAQ was 1.0 (IQR: 0, 5.0). Further characteristics of the included cases are presented in Table 1.

**Table 1:** Characteristics of the study population and comparison between healthy controls, and those with MCI and dementia

***Characteristic***	***N***	***Overall****,* *N* = *2,216*^*a*^	***Healthy controls*** *N* = *786*^*a*^	***MCI*** *N* = *1,060*^*a*^	***Dementia*** *N* = *370*^*a*^	***P-value***^*2*^
***Age (years)***	2,216	73 (68, 78)	72 (68, 77)	73 (68, 78)	75 (70, 80)	** < 0.001**
***Sex***	2,216					** < 0.001**
* Female*		1,045 (47%)	440 (56%)	442 (42%)	163 (44%)	
* Male*		1,171 (53%)	346 (44%)	618 (58%)	207 (56%)	
***Educational level (years)***	2,216	16.00 (14.00, 18.00)	16.00 (15.00, 18.00)	16.00 (14.00, 18.00)	16.00 (13.00, 18.00)	** < 0.001**
***Marital status***	2,216					** < 0.001**
* Currently married*		1,673 (75%)	547 (70%)	815 (77%)	311 (84%)	
* Currently not married or unknown*		543 (25%)	239 (30%)	245 (23%)	59 (16%)	
***Home***	2,216					0.8
* House or appartment*		2,101 (95%)	748 (95%)	1,006 (95%)	347 (94%)	
* Retirement or nursing institution*		76 (3.4%)	26 (3.3%)	34 (3.2%)	16 (4.3%)	
* Other*		39 (1.8%)	12 (1.5%)	20 (1.9%)	7 (1.9%)	
***Racial profile***	2,216					** < 0.001**
* White*		1,952 (88%)	651 (83%)	963 (91%)	338 (91%)	
* Black*		167 (7.5%)	92 (12%)	56 (5.3%)	19 (5.1%)	
* Other*		97 (4.4%)	43 (5.5%)	41 (3.9%)	13 (3.5%)	
***APOE ε4 status***	2,031					** < 0.001**
* 0 allele*		1,095 (54%)	488 (70%)	495 (50%)	112 (32%)	
* 1 allele*		735 (36%)	188 (27%)	378 (39%)	169 (48%)	
* 2 alleles*		201 (9.9%)	22 (3.2%)	108 (11%)	71 (20%)	
* Missing values*		185	88	79	18	
***ADAS***_***13***_*** total score***	2,216	14 (9, 22)	8 (5, 12)	16 (11, 21)	29 (24, 34)	** < 0.001**
***MMSE total score***	2,216	28.00 (26.00, 29.00)	29.00 (29.00, 30.00)	28.00 (26.00, 29.00)	23.00 (21.00, 25.00)	** < 0.001**
***CDR-SB***	2,216	1.00 (0.00, 2.00)	0.00 (0.00, 0.00)	1.50 (1.00, 2.00)	4.50 (3.50, 5.00)	** < 0.001**
***FAQ total score***	2,211	1.0 (0.0, 5.0)	0.0 (0.0, 0.0)	1.0 (0.0, 5.0)	13.0 (8.0, 18.0)	** < 0.001**
* Missing values*		5	0	4	1	
***GDS total score***	2,216	1.00 (0.00, 2.00)	0.00 (0.00, 1.00)	1.00 (1.00, 3.00)	1.00 (1.00, 3.00)	** < 0.001**
***BMI***	2,216	26.3 (23.9, 29.3)	26.7 (24.1, 30.0)	26.3 (24.0, 29.2)	25.4 (23.1, 28.0)	** < 0.001**
***Cholesterol levels (mg/dL)***	2,216	191 (165, 220)	189 (165, 217)	191 (165, 221)	193 (167, 224)	0.4
***Triglyceride levels (mg/dL)***	2,216	120 (87, 171)	116 (85, 164)	121 (86, 177)	124 (93, 171)	0.078
***Sleep disorders at baseline***	2,216	357 (16%)	80 (10%)	193 (18%)	84 (23%)	** < 0.001**
***Sleep disorders at 12 months***	1,845	327 (18%)	56 (8.7%)	200 (22%)	71 (23%)	** < 0.001**
* Missing values*		371	141	163	67	

*ADAS*_*13*_ Alzheimer’s Disease Assessment Score – 13 items, *APOE ε4* Apolipoprotein E ε4, *BMI* Body-Mass Index (Weight in Kg / (Height in m)^2^), *CDR- SB* Clinical Dementia Rating Scale—sum of boxes, *FAQ* Functional Activities Questionnaire, *GDS* Geriatric Depression Scale, *MCI* Mild Cognitive Impairment, *MMSE* Mini-Mental Status Examination

^a^Median (IQR); n (%)

^2^Kruskal–Wallis rank sum test; Pearson’s Chi-squared test

### Comparison between healthy controls, and those with MCI and dementia

Cholesterol levels ranged from 74 to 476 mg/dL, the median in the total population was 191 mg/dL (IQR: 165, 220), 189 mg/dL (IQR: 165, 217) in HC, 191 mg/dL (IQR: 165, 221) in the MCI group, and 193 mg/dL (IQR: 167, 224) in those with dementia. No statistically significant difference was found between diagnosis groups (*P*-value = 0.4). The median Triglyceride level was 120 mg/dL (IQR: 87, 171) in the main population, and ranges between 32 and 2084 mg/dL. No statistically significant difference was found between diagnostic groups (116, 121, and 124 mg/dL, respectively, *P*-value = 0.078). The median BMI of the main population was 26.3 (IQR: 23.9, 29.3), ranging between 17.14 and 51.75, with 19 (0.86%) classified as underweight, 796 (35.92%) as healthy weight, 930 (41.97%) as overweight, and 471 (21.25%) as obese. There was a statistically significant difference between BMI medians observed in different groups (26.7 in HC, 26.3 in MCI, and 25.4 in the dementia group, *P*-value < 0.001).

### Comparison between study participants with and without dyslipidemia

**Fig. 2:**
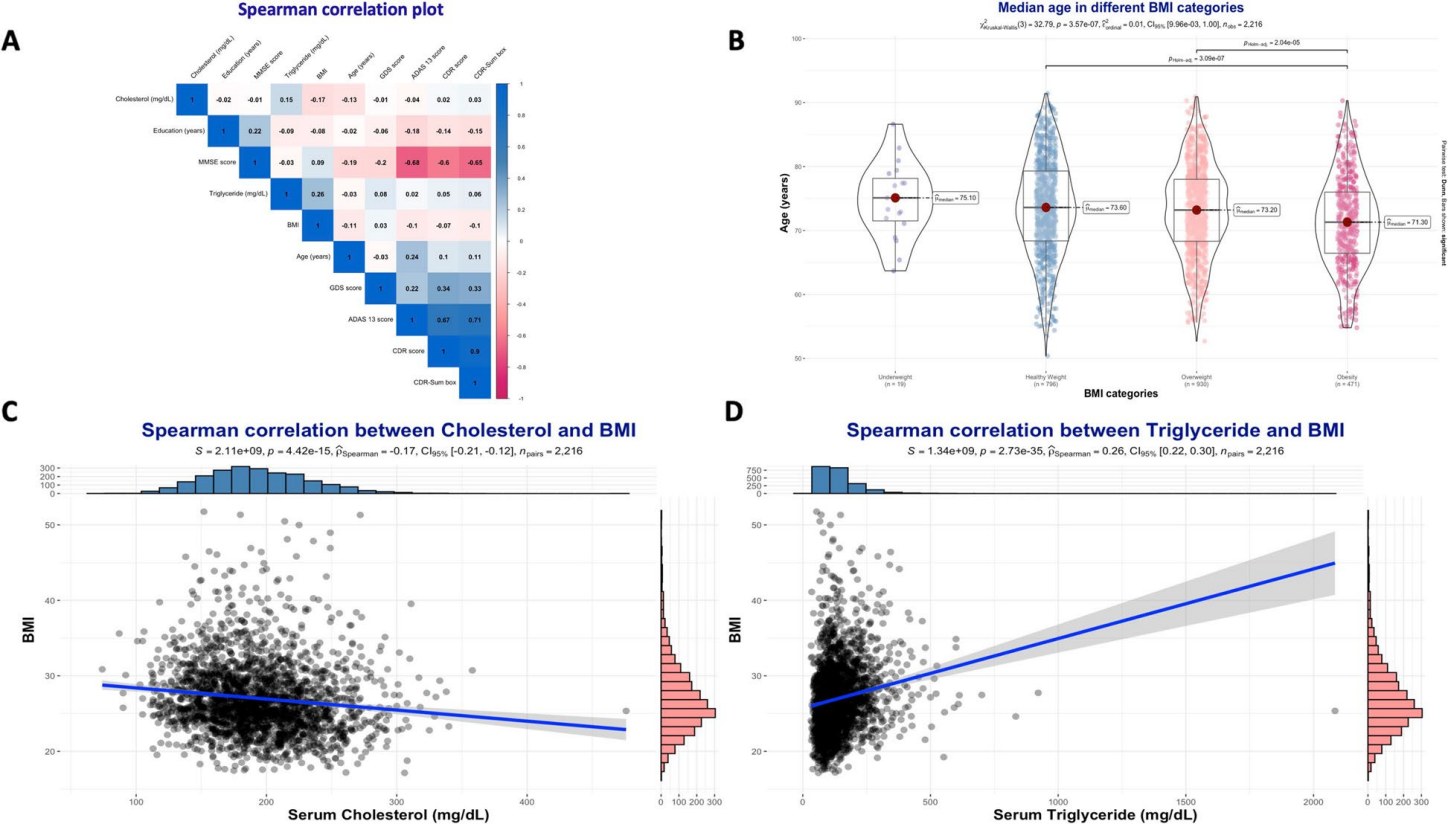
BMI categories and correlation analyses. **A** Spearman correlation plot. **B** Median age across different BMI categories. **C** Spearman correlation between Cholesterol and BMI. **D** Spearman correlation between Triglyceride and BMI

Based on clinical cutoff values, hypercholesterolemia was diagnosed in 920 cases (41.52%), and hypertriglyceridemia in 725 cases (32.72%). Details on the differences between groups are presented in Table 2. BMI was significantly higher in the group without hypercholesterolemia than in those with (26.7 vs. 25.5, *P*-value < 0.001). In contrast, BMI was higher in those with hypertriglyceridemia than in those without (25.7 vs. 27.5, *P*-value < 0.001). Very weak correlations were found between hypercholesterolemia and hypertriglyceridemia with neurocognitive scores (Fig. 2A). BMI showed a decreasing tendency with age, and cases with obesity were significantly younger than those with overweight and those with a healthy weight (Fig. 2B). Furthermore, BMI was negatively correlated with serum Cholesterol levels, and positively correlated with serum Triglyceride levels (Fig. 2C and D).

**Table 2:** Comparison between study participants with and without dyslipidemia

***Characteristic***	** Hypercholesterolemia **			** Hypertriglyceridemia **		
	**Normal** **(< 200 mg/dL)** N = 1,296^1^	**High** **(≥ 200 mg/dL)** N = 920^1^	***P*-value**^2^	**Normal** **(< 150 mg/dL)** N = 1,491^1^	**High** **(≥ 150 mg/dL)** N = 725^1^	***P*-value**^2^
***Age (years)***	74 (69, 79)	72 (67, 78)	** < 0.001**	73 (68, 78)	73 (67, 78)	0.3
***Sex***			** < 0.001**			0.4
* Female*	453 (35%)	592 (64%)		713 (48%)	332 (46%)	
* Male*	843 (65%)	328 (36%)		778 (52%)	393 (54%)	
***Main cognitive diagnosis***			0.6			0.3
* Healthy controls*	465 (36%)	321 (35%)		543 (36%)	243 (34%)	
* MCI*	623 (48%)	437 (48%)		698 (47%)	362 (50%)	
* Dementia*	208 (16%)	162 (18%)		250 (17%)	120 (17%)	
***Educational level (years)***	16.00 (14.00, 18.00)	16.00 (14.00, 18.00)	0.4	16.00 (14.00, 18.00)	16.00 (14.00, 18.00)	** < 0.001**
***Marital status***			**0.004**			0.6
* Currently married*	1,007 (78%)	666 (72%)		1,131 (76%)	542 (75%)	
* Currently not married or unknown*	289 (22%)	254 (28%)		360 (24%)	183 (25%)	
***Home***			0.4			0.5
* House or appartment*	1,230 (95%)	871 (95%)		1,419 (95%)	682 (94%)	
* Retirement or nursing institution*	47 (3.6%)	29 (3.2%)		47 (3.2%)	29 (4.0%)	
* Other*	19 (1.5%)	20 (2.2%)		25 (1.7%)	14 (1.9%)	
***Racial profile***			0.6			** < 0.001**
* White*	1,140 (88%)	812 (88%)		1,294 (87%)	658 (91%)	
* Black*	95 (7.3%)	72 (7.8%)		137 (9.2%)	30 (4.1%)	
* Other*	61 (4.7%)	36 (3.9%)		60 (4.0%)	37 (5.1%)	
***APOE ε4 status***			**0.024**			0.3
* 0 allele*	663 (56%)	432 (51%)		727 (53%)	368 (55%)	
* 1 allele*	411 (35%)	324 (38%)		489 (36%)	246 (37%)	
* 2 alleles*	104 (8.8%)	97 (11%)		145 (11%)	56 (8.4%)	
* Missing values*	118	67		130	55	
***ADAS***_***13***_*** total score***	14 (9, 21)	14 (8, 22)	0.059	14 (9, 21)	14 (9, 22)	0.4
***MMSE total score***	28.00 (26.00, 29.00)	28.00 (26.00, 30.00)	> 0.9	28.00 (26.00, 29.00)	28.00 (26.00, 29.00)	0.2
***CDR-SB***	1.00 (0.00, 2.00)	1.00 (0.00, 2.50)	0.4	1.00 (0.00, 2.00)	1.00 (0.00, 2.50)	0.081
***FAQ total score***	1.0 (0.0, 5.0)	1.0 (0.0, 5.0)	0.7	0.0 (0.0, 5.0)	1.0 (0.0, 6.0)	**0.028**
* Missing values*	1	4		4	1	
***GDS total score***	1.00 (0.00, 2.00)	1.00 (0.00, 2.00)	0.7	1.00 (0.00, 2.00)	1.00 (0.00, 2.00)	** < 0.001**
***BMI***	26.7 (24.4, 29.8)	25.5 (23.1, 28.7)	** < 0.001**	25.7 (23.4, 28.6)	27.5 (25.1, 30.9)	** < 0.001**
***Cholesterol (mg/dL)***	170 (151, 184)	225 (212, 246)	** < 0.001**	187 (162, 216)	197 (172, 227)	** < 0.001**
***Triglyceride (mg/dL)***	115 (84, 162)	127 (92, 183)	** < 0.001**	98 (76, 120)	203 (172, 255)	** < 0.001**
***Sleep disorders at baseline***	206 (16%)	151 (16%)	0.7	216 (14%)	141 (19%)	**0.003**
***Sleep disorders at 12 months***	188 (17%)	139 (18%)	0.7	199 (16%)	128 (21%)	**0.025**
* Missing values*	215	156		270	101	

*ADAS13* Alzheimer’s Disease Assessment Score – 13 items, *APOE ε4 *Apolipoprotein E ε4,* BMI *Body-Mass Index (Weight in Kg / (Height in m)^2^), *CDR- SB *Clinical Dementia Rating Scale—sum of boxes, *FAQ *Functional Activities Questionnaire, *GDS *Geriatric Depression Scale,* MCI *Mild Cognitive Impairment, *MMSE *Mini-Mental Status Examination

^1^Median (IQR); n (%)

^2^Wilcoxon rank sum test; Pearson’s Chi-squared test

**Fig. 3:**
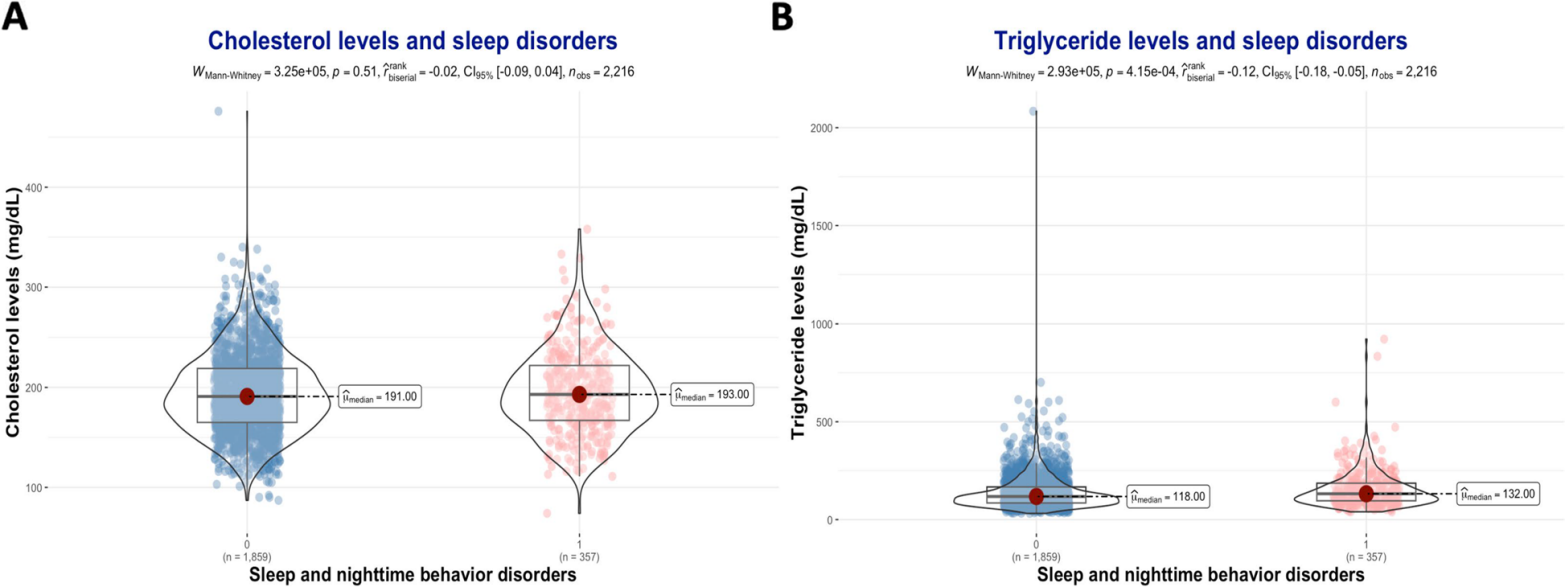
Dyslipidemia and sleep disorders. **A** Cholesterol levels and sleep disorders. **B** Triglyceride levels and sleep disorders

There was no significant difference in Cholesterol levels between those with SNBD at baseline and those without (16% vs. 16%, *P*-value = 0.7). Similar results were also observed at 12 months (17% vs. 18%, *P-*value = 0.7). Thus, there were more cases of SNBD in the hypertriglyceridemia group than in those with normal triglyceride (19% vs. 14%, *P*-value = 0.003). Similarly, 21% of cases of SNBD at 12 months had hypertriglyceridemia and 16% had normal triglyceride levels at baseline (*P*-value = 0.025) (Table 2). Cases with SNBD had higher serum Cholesterol levels than those without SNBD but the difference was not statistically significant (193 vs. 191 mg/dL, *P*-value = 0.51) (Fig. 3A). In contrast, cases with SNBD had significantly higher serum Triglyceride levels than those without (132 vs. 118 mg/dL, *P*-value < 0.001) (Fig. 3B).

### Association between sleep and nighttime behavior and dyslipidemia

At baseline, participants with hypercholesterolemia had 4% higher odds of SNBD but the results were not statistically significant (OR = 1.04, 95% *CI*: 0.83, 1.31, *P*-value = 0.744). No statistical significance was observed after adjustment for confounding factors.

Those with hypertriglyceridemia levels had 43% higher odds of SNBD at baseline (OR = 1.43, 95% *CI*: 1.13, 1.80, *P*-value = 0.003). The results remained statistically significant after adjustment for confounding factors (adj. OR = 1.36, 95% *CI*: 1.06, 1.74, *P*-value = 0.016) and after adding BMI to the adjusted model (BMI-adj. OR = 1.29, 95% *CI*: 1.00, 1.66, *P-*value = 0.048). BMI and particularly obesity were significantly associated with higher odds of SNBD at baseline. No significant associations were found between hypercholesterolemia and hypertriglyceridemia with incident SNBD at 12 months of follow-up. Detailed results of the logistic regression were reported in Table 3.

**Table 3:** Association between sleep and nighttime behavior and dyslipidemia at baseline and 12 months follow-up

*Characteristic*	*Model 1*				*Model 2*				*Model 3*			
	***N***	***Event***	***OR***^*a*^* (****95% CI***^*a*^*)*	***P-value***	***N***	***Event***	***OR***^*a*^* (****95% CI***^*a*^*)*	***P-value***	***N***	***Event***	***OR***^*a*^* (****95% CI***^*a*^*)*	***P-value***
***Sleep disorders at baseline***												
*** Cholesterol***	2,216	357		0.744	2,031	335		0.412	2,031	335		0.571
* Normal (*< *200 mg/dL)*			—				—				—	
* High (*≥ *200 mg/dL)*			1.04 (0.83, 1.31)				0.90 (0.70, 1.16)				0.93 (0.72, 1.20)	
*** Triglyceride***	2,216	357		**0.003**	2,031	335		**0.016**	2,031	335		**0.048**
* Normal (*< *150 mg/dL)*			—				—				—	
* High (*≥ *150 mg/dL)*			1.43 (1.13, 1.80)				1.36 (1.06, 1.74)				1.29 (1.00, 1.66)	
*** BMI (continuous value)***	2,216	357	1.03 (1.00, 1.05)	**0.030**	2,031	335	1.03 (1.01, 1.06)	**0.008**			—	
***BMI categories***	2,216	357		0.095	2,031	335		**0.029**			—	
* Healthy Weight*			—				—				—	
* Underweight*			1.12 (0.26, 3.43)	0.857			0.95 (0.21, 2.99)	0.934			—	
* Overweight*			1.12 (0.86, 1.47)	0.391			1.14 (0.86, 1.51)	0.371			—	
* Obesity*			1.47 (1.09, 1.99)	**0.012**			1.65 (1.18, 2.29)	**0.003**			—	
***Incident sleep disorders at 12 months follow-up***												
*** Cholesterol***	1,550	187		0.719	1,468	178		0.641	1,468	178		0.706
* Normal (*< *200 mg/dL)*			—				—				—	
* High (*≥ *200 mg/dL)*			1.06 (0.77, 1.44)				1.08 (0.77, 1.52)				1.07 (0.76, 1.50)	
*** Triglyceride***	1,550	187		0.066	1,468	178		0.167	1,468	178		0.122
* Normal (*< *150 mg/dL)*			—				—				—	
* High (*≥ *150 mg/dL)*			1.35 (0.98, 1.85)				1.27 (0.90, 1.76)				1.31 (0.93, 1.83)	
*** BMI (continuous value)***	1,550	187	0.98 (0.95, 1.01)	0.202	1,468	178	0.98 (0.95, 1.02)	0.417			—	
***BMI categories***	1,550	187		**0.046**	1,468	178		0.090			—	
* Healthy Weight*			—				—				—	
* Underweight*			4.81 (1.23, 16.4)	**0.014**			4.15 (1.04, 14.6)	**0.030**			—	
* Overweight*			1.34 (0.95, 1.90)	0.101			1.35 (0.93, 1.95)	0.114			—	
* Obesity*			0.97 (0.61, 1.51)	0.891			1.02 (0.62, 1.66)	0.941			—	

Model 1: crude logistic regression analysis

Model 2: adjusted for age, sex, racial profile, educational level, cognition-related main diagnosis, geriatric depression scale total score, APOE ε4 status

Model 3: model 2 + BMI

^a^*OR* Odds Ratio, *CI* Confidence Interval, *BMI* Body Mass Index

## Discussion

This study explored the association between dyslipidemia based on pathological Cholesterol or Triglyceride levels at baseline, and SNBD reported by the study partner of included older participants at two time points.

The main outcome was the significant association between hypertriglyceridemia and sleep disorders in the cross-sectional analysis at baseline, even after adjusting for age, sex, racial profile, educational level, GDS total score, APOE ε4, cognition-related main diagnosis, and BMI.

### Sleep duration and dyslipidemia

Although insomnia is a well-recognized risk factor for cardiovascular and metabolic complications [24, 25], the significant association between dyslipidemia and sleep disorders extends beyond sleep deprivation. Several population-based studies described a U-shaped association between sleep duration and serum lipid levels, where both short and long sleep durations were significantly associated with dyslipidemia. In people with longer sleep durations, high Triglyceride levels were commonly described [26–28].

The novelty in the current study was the analysis of associations based on clinical cutoff values defining hypercholesterolemia or hypertriglyceridemia, and a binary outcome defining the existence or not of SNBD. Information on sleep hours was not part of the ADNI investigations, and the focus was SNBD as an outcome rather than sleep duration as an exposure.

### Sleep quality and dyslipidemia

In addition to the metabolic effect associated with the quantitative dimension of sleep, lipid levels might also be modulated by the subjective sleep quality [14]. Difficulty in maintaining sleep and excessive daytime sleepiness increased the odds of metabolic syndrome in the elderly, independently of obesity and snoring [29]. Moreover, the consumption of sleep medication, a biomarker of sleep disorder severity, showed a significant association with elevated low-density lipoprotein-cholesterol (LDL-C) [30]. Neither LDL-C nor heigh-density lipoprotein-C (HDL-C) were reported in the main ADNI laboratory data.

### Sleep dysregulation and dyslipidemia

Night work, sleep debt, and social jetlag present further risk factors impairing sleep homeostasis and are associated with dyslipidemia and cardiovascular risks [31, 32]. Amongst 5,813 study participants from the Korean National Health and Nutrition Examination Survey (2013–2016), males exercising night work had 53% higher odds of being diagnosed with dyslipidemia. Compared to day-working male participants, male night workers who slept less than six hours and those who skipped meals had significantly higher odds of dyslipidemia [33].

### Obstructive sleep apnea and dyslipidemia

The associations between OSA and metabolic syndrome [34, 35], as well as the association between OSA and triglyceride-glucose index, a biomarker of insulin resistance [36], are well-described in the literature. Moreover, novel lipid indices, mainly lipid accumulation product (LAP), visceral adiposity index (VAI), and atherogenic index of plasma (AIP) were found to be higher in people with OSA than controls [37]. Thus, independent of the OSA diagnosis, frequent snoring was also associated with dyslipidemia and predicted linearly higher levels of Triglyceride in a large population study [38].

### Associations in younger patients

The focus of the current study was older adults with and without cognitive decline. But, the sleep-lipid association was described in younger age groups as well. Higher Triglyceride levels were also reported in adolescents with longer sleep hours [39], and accelerometry-based sleep clustering also showed that male adolescents with sleep irregularities had significantly higher Triglyceride levels [40].

### Longitudinal studies

The second outcome was the absence of a significant association between dyslipidemia at baseline and incident SNBD over a 12-month follow-up period.

While cross-sectional design is inadequate in inferring the causal relationship between sleep and dyslipidemia, longitudinal studies in older adults showed a bidirectional association between sleep duration and blood lipids. Total Cholesterol, LDL-C, HDL-C, and Triglycerides showed different temporal relationships with sleep duration. BMI and age were significant effect modifiers in this association [41]. In a longitudinal population-based cohort of healthy adults, short sleep duration increased the risk of metabolic syndrome, particularly hypertriglyceridemia by 9%. In comparison, long sleep duration decreased the risk of hypertriglyceridemia by 11% [42].

### Mechanisms

The significant association between sleep disorders and higher Triglyceride levels might be explained by concomitant stress, frustration, and consequently eating irregularities. Stress and anxiety are associated with both sleep and eating disorders [43]. First, the association between sleep and stress is bidirectional; an increased emotional stress level might lead to sleep irregularities or insomnia, and sleep disorders might cause higher stress and frustration [44]. Sleep is crucial for emotion regulation and vice versa [45, 46]. Second, sleep disturbance interacts with hormone secretion and eating disorders [47]. Studies have shown that after sleep deprivation, people express eating and appetite dysregulation [48, 49]. Ghrelin, Leptin, and Adiponectin secretion patterns present a mediating effect on the association between sleep duration, and metabolic syndrome [50, 51]. Further, autoimmunity and neuroinflammation are relevant mechanisms involved in the homeostatic dysregulation associated with sleep disorders, and higher inflammatory biomarkers might further impair metabolic function and energy regulation [52]. Finally, a genetic predisposition, particularly Apolipoprotein genes, might infer the association between sleep and dyslipidemia [53].

The sleep-lipid association was controversially discussed. Linear models seem insufficient to explain the association [40]. Moreover, published results were mainly different depending on the adjustment model used in the analysis. Noteworthy, adjusting for BMI and OSA led to the loss of the statistical significance of the association between sleep duration and quality on one side, and serum Triglyceride and hepatic Triglyceride content on another side [16]. It is known that BMI and OSA infer sleep quality and lipid levels and therefore might present a confounding effect on the path between sleep and dyslipidemia. This contradicts current results since associations remained statistically significant even after adjusting for BMI, and the questions on which the analysis of SNBD was based have mainly a behavioral aspect without considering respiratory symptoms.

### Strengths

The major strength of the study is the large number of included cases with high-quality and complete data. The study’s inclusion criteria were very restrictive, and only complete cases were considered for the analysis, lowering the bias risk and giving the data a strong analytical value. Older adults tend to be less represented in epidemiological and molecular studies. Therefore, restricting the included population to advanced age groups is a further strength. Older adults have a particularly higher risk of multimorbidity and polymedication. Reducing the risk of one factor might improve the prognosis of other health conditions. This helps lower health costs, in addition to reducing morbidity and mortality risks. Furthermore, the combination of a cross-sectional and longitudinal design in the analyses allowed a better evaluation of the association’s predictive value.

This is the first study exploring the association between informant-reported SNBD and dyslipidemia. Most of the published data studied either OSA or self-reported sleep disorders. In older populations, the self-awareness of sleep quality might be impaired, and this study presents a further strength related to the partner-provided information on sleep-related behavior disorders.

A further strength is related to the fact that the study was based on results obtained from fasting blood. However, a study on the association between sleep disturbances and Triglyceride levels in adolescents showed that the results were not affected by the fasting status of study participants and were statistically significant before and after stratifying by fasting during blood sampling [40].

### Limitations

Despite the interesting findings reported in this study, it is important to acknowledge some limitations.

The first limitation is related to the absence of information on LDL-C and HDL-C levels; both of which are relevant biomarkers of metabolic syndrome and dyslipidemia; but were not assessed in the accessible main laboratory ADNI data. A further limitation is related to the lack of information on comorbid non-alcoholic fatty liver disease (NAFLD) or sarcopenia. Both of them are associated with sleep disorders [54, 55]. In this study, BMI was considered a solid surrogate biomarker for NAFLD [56, 57], nutrition, and physical activity and was introduced to the adjusted models. Although BMI predicted independently sleep disorders in the univariable models, incorporating BMI in the adjusted models (Model 3) of Cholesterol and then of Triglyceride did not impact the overall statistical significance of the results. Noteworthy, the association between hypertriglyceridemia and SNBD was not influenced by BMI as covariable and remained statistically significant.

The second limitation is the absence of subjective data such as sleep duration or detailed polysomnographic measurements (Gold Standard). Furthermore, neither information on the objective description of the sleep quality reported by study participants, nor the presence or absence of OSA were included. While those explorations are of high interest and might have enriched diagnostic methods and information used in the current study, they were largely explored in published data. The informant-based evaluation was favored, particularly because of its relevance in older adults with cognitive impairment who might lack to some extent self-awareness and tend to over- or underestimate their sleep duration. The questionnaire was oriented toward sleep behavior disorders rather than breathing disorders and associated disturbances.

The third limitation is the lack of information on whether study participants were under lipid-regulating medications. Although this information could be relevant for evaluating the overall prevalence of dyslipidemia in the included sample, the main study objective was to investigate the association between pathological lipid levels at baseline as predicting variable and concomitant sleep disorders, independently of the potential medication effect.

Finally, the study is of predictive value and does not allow drawing causal inferences from the association between dyslipidemia and SNBD.

## Conclusions

Hypertriglyceridemia, but not hypercholesterolemia, was associated with SNBD in older adults at baseline. This association was independent of BMI. However, none of the dyslipidemia forms did predict incidental cases of SNBD over a follow-up period of 12 months. The current study emphasizes the importance of systematic screening of sleep disorders in older patients and its adapted management in mitigating metabolic risks and preventing related cardiovascular complications. Informant-based interviews are helpful and provide complementary information on sleep disorders in older individuals with cognitive impairment.

## Supplementary Information

Supplementary Material 1.

## Data Availability

All data available at https://adni.loni.usc.edu.
