# Supplementary material for: Sleep and nighttime behavior disorders in older adults: associations with hypercholesterolemia and hypertriglyceridemia at baseline, and a predictive analysis of incident cases at 12 months follow-up
Source: Lipids Health Dis. 2024 Sep 28;23:320. doi: 10.1186/s12944-024-02302-x (PMC11439313; doi:10.1186/s12944-024-02302-x)
Supplement: Supplementary file 1 — Supplementary Material 1. [file 12944_2024_2302_MOESM1_ESM.pdf]

Sleep and nighttime behavior disorders in older adults: associations with hypercholesterolemia and hypertriglyceridemia at baseline and a prediction analysis of incidental cases at 12 months follow-up

By Asma Hallab

WORD COUNT	7394	TIME SUBMITTED	12-SEP-2024 10:13AM
		PAPER ID	111660166

Sleep and nighttime behavior disorders in older adults: associations with hypercholesterolemia and hypertriglyceridemia at baseline and a prediction analysis of incidental cases at 12 months follow-up

Asma Hallab [a,b,c#], for the Alzheimer's Disease Neuroimaging Initiative*

Affiliations

a- Biologie Intégrative et Physiologie – Parcours Neurosciences Cellulaires et Integrées. Faculté des Sciences et Ingénierie. Campus Pierre et Marie Curie. Sorbonne Université, Paris, France.

b- Pathologies du Sommeil. Faculté de Médecine. Hopital Universitaire Pitié-Salpêtrière. Sorbonne Université, Paris, France.

c- Charité - Universitätsmedizin Berlin, Corporate member of Freie Universität Berlin and Humboldt-Universität zu Berlin. Institute of Public Health. Berlin, Germany.

Number of words in the abstract: 275

Number of words in the manuscript: 3573

Number of tables: 3

Number of figures: 3

[#]**Corresponding author:** Dr. med. Dr. Asma Hallab. Charité Universitätsmedizin – Berlin. Charitéplatz 1, 10117 Berlin – Germany. asma.hallab@charite.de.

https://orcid.org/0000-0002-3901-7980

Abstract

Introduction

Sleep disorders, particularly insomnia and obstructive sleep apnea, are associated with dyslipidemia in the general population. The study's aim was to explore the association between pathological Cholesterol and Triglyceride levels, and sleep and nighttime behavior disorders (SNBD) in older adults, whether they might predict SNBD onset, and to emphasize the role of body mass index (BMI) in this association.

Methods

Alzheimer's Disease Neuroimaging Initiative (ADNI) population with complete Cholesterol, Triglyceride, SNBD, and neurocognitive data were included. Logistic regression was performed to study the association between hypercholesterolemia, hypertriglyceridemia, and SNBD at baseline and 12 months. Relevant confounders, particularly BMI, were adjusted for.

Results

Among the 2,216 included cases, 1,045 (47%) were females, and the median age was 73 years (IQR: 68, 78). At baseline, 357 (16%) had SNBD and 327 (18%) at 12 months; 187 of them were incident cases.

There were more cases of baseline SNBD in the hypertriglyceridemia group than in those without (19% vs. 14%, *P*-value=0.003). Similarly, more follow-up SNBD cases had hypertriglyceridemia at baseline (21% vs. 16%, *P*-value=0.025). SNBD cases at baseline had significantly higher serum Triglyceride levels than those without (132 vs. 118mg/dL, *P*-value<0.001).

Only hypertriglyceridemia was significantly associated with baseline SNBD (crude OR=1.43, 95%*CI*: 1.13,1.80, *P*-value=0.003), even after adjustment for confounding factors (adj.

OR=1.36, 95%*CI*: 1.06,1.74, *P*-value=0.016) and (BMI-adj. OR=1.29, 95%*CI*: 1.00,1.66, *P*-value=0.048). None of the dyslipidemia forms did predict incident cases at 12 months.

Conclusions

Hypertriglyceridemia, but not hypercholesterolemia, was associated with higher odds of SNBD. The association was independent of BMI. None of the dyslipidemia forms predicted incidental SNBD over 12 months. Sleep disorders should motivate a systematic screening of dyslipidemia in older adults and vice versa.

Keywords: Sleep, Aging, Dyslipidemia, Triglyceride, Cholesterol, BMI.

1. Introduction

Sleep disorders represent a large spectrum of symptoms defining an alteration of sleep quality, structure, chronobiology, duration, and associated breathing and movement disorders. (1) Insomnia and sleep obstructive apnea syndrome have a high prevalence worldwide; both are associated with cardiovascular and neuropsychiatric risk factors and affected persons are exposed to higher morbidity and mortality rates. (2-5) The incidence of sleep disorders increases with age. In addition to the physiological decrease in sleep hours during the aging process; neurodegeneration, neuroendocrine, and sleep disorders define a more complex bidirectional association. (6) Old patients with sleeping disorders, particularly insomnia, have higher risks of cognitive decline, and those with cognitive impairment are more susceptible to progressing into dementia. (7-9) Moreover, elderly patients with neurocognitive disorders are at higher risk of experiencing neurodegeneration-related sleep behavior and movement disorders. (10)

The relationship between sleep disorders and metabolic and cardiovascular pathologies is largely reported in the literature. (11, 12) Defined as the association between diabetes, dyslipidemia, hypertonia, and visceral adiposity, the metabolic syndrome is a well-established risk factor for cardiovascular disorders and is related to higher morbidity and mortality rates. (13) Most published studies evaluated sleep disorders quantitatively based on sleep duration or qualitatively depending on the subjective perception of sleep quality. (14-17) Studies on sleep disorders objectified by the study partner of older patients and their association with dyslipidemia are rare. Moreover, there is limited data on whether dyslipidemia might predict prospectively sleep disorders. It is also unclear how much this association depends on body mass index (BMI), particularly in older adults. The overall study question was whether hypercholesterolemia or hypertriglyceridemia, independent of BMI, are associated with sleep and nighttime behavior disorders (SNBD) in advanced age groups.

The aims of this study were (1) to explore the association between dyslipidemia and informant-perceived SNBD in older adults at baseline, and (2) to evaluate whether dyslipidemia at baseline might predict incidental cases of SNBD over 12 months of follow-up.

2. Methods

This manuscript has been prepared and reported according to STROBE guidelines. (18)

- **Study population**

The studied population is part of the Alzheimer's Disease Neuroimaging Initiative (ADNI) cohort, from which only cases with complete data required for the current analysis were included. Dr Michael W. Weiner is ADNI's principal investigator. ADNI is a non-interventional longitudinal study. Study participants are older adults recruited at 59 centers around the

United States and Canada, who underwent an observational follow-up where biological, genetic, neuroimaging, and neuropsychiatric information was assessed at several time points. Participants from different phases (ADNI 1, go, 2, and 3) were eligible for the current analysis. The study was performed according to the Declaration of Helsinki and ethical approval was obtained from the Internal Reviewing Board corresponding to each participating site. Written consent was obtained from all ADNI study participants. Data, ethical approval, enrollment, and protocols can be found at https://adni.loni.usc.edu.

- **Cholesterol and Triglyceride measurements**

Serum Cholesterol and Triglyceride levels were assessed at baseline and mainly reported in mg/dL. Laboratory normal ranges were 0 - 199 mg/dL for Cholesterol and 0 - 149 mg/dL for Triglyceride. Defect and duplicated measurements were checked for each individual and removed based on the date and time of the reported result. Serum levels corresponding to 200 mg/dL for Cholesterol and 150 mg/dL for Triglyceride are largely recognized clinical cutoff values for hypercholesterolemia and hypertriglyceridemia, respectively. (19, 20) Owing to the larger use of mg/dL as a unit worldwide, values in mmol/L were converted to mg/dL:

$$\text{Cholesterol (mg/dL)} = \text{Cholesterol (mmol/L)} \times 38.67$$

$$\text{Triglycerides (mg/dL)} = \text{Triglycerides (mmol/L)} \times 88.57$$

- **Sleep and nighttime behavior disorders**

The assessment of SNBD was based on the neuropsychiatric inventory questionnaire (NPI/NPI-Q) filled by study partners of included participants. (21, 22) The item related to sleep disorders in NPI/NPI-Q covered the following questions:

"Does the patient have difficulty sleeping (do not count as present if the patient simply gets up once or twice per night only to go to the bathroom and falls back asleep immediately)? Is he/she up at night? Does he/she wander at night, get dressed, or disturb your sleep? "

If this question was answered with yes, details on the following questions were then collected:

1. "Does the patient have difficulty falling asleep?"
2. "Does the patient get up during the night?"
3. "Does the patient wander, pace, or get involved in inappropriate activities at night?"
4. "Does the patient awaken you during the night?"
5. "Does the patient wake up at night, dress, and plan to go out, thinking that it is morning and time to start the day?"
6. "Does the patient awaken too early in the morning, earlier than was his/her habit?"
7. "Does the patient sleep excessively during the day?"
8. "Does the patient have any other nighttime behaviors that bother you and we haven't talked about?"

- **Cognitive tests**

Cognition was assessed based on the Alzheimer's Disease Assessment Score with 13 items ($ADAS_{13}$), Mini-Mental Status Examination (MMSE) total score, Clinical Dementia Rating (CDR) total score, CDR-sum of boxes (CDR-SB), and Functional Activities Questionnaire (FAQ) total score. Moreover, depression symptoms were reported based on the total Geriatric Depression Scale (GDS) score. People with severe depression were initially excluded from ADNI and therefore included participants are either non- or mildly depressed. The included cases were either healthy controls (HC), participants with mild cognitive impairment (MCI), or dementia.

- **Body-mass index**

Weight and Height data were checked to ensure the plausibility of the values and units. Weight at baseline was considered and converted to "Kilogram" when the unit of measurement was "Pounds":

Kilograms= 2.20462 * Pounds

Similarly, height was converted to "Meters" when the unit of measurement was "Inches":

Meters= 0.0254 * Inches

BMI was calculated based on the formula weight (Kg) /Height (m) ^{2}. (23)

- **Inclusion criteria**

After excluding 142 participants without Cholesterol or Triglyceride measurements, 27 without complete NPI/NPI-Q, 21 missing total $ADAS_{13}$ and one missing GDS score at baseline, nine missing baseline main diagnosis, and four missing complete demographic data (age), four missing weight or height at baseline and six have erroneous measurements or units, a total of 2,216 study participants were included in the analysis (fig. 1A). Among those 371 were lost to follow-up at 12 months (fig. 1B).

- **Statistical analysis**

The statistical analysis was performed by RStudio version 2024-04. Continuous data was reported as median (Inter-quartile range (IQR)) and count data as number (percentage (%)). Kruskal-Wallis rank sum test and Pearson's Chi-squared test were performed to compare groups and for each analysis, the *P*-value was reported. Spearman correlation between serum Cholesterol, Triglyceride, age, and scores of cognitive tests, was performed and correlation coefficients were reported. The association between SNBD and dyslipidemia at baseline was evaluated using logistic regression with SNBD as a dependent binary variable, and

dyslipidemia as an independent binary variable. Models were adjusted for age, sex, racial profile, educational level, GDS total score, APOE ε4 status, main diagnosis related to cognitive status, and BMI, as follows:

Model 1: crude logistic regression analysis,

Model 2: adjusted for age, sex, racial profile, educational level, cognition-related main diagnosis, geriatric depression scale total score, APOE ε4 status,

Model 3: model 2 + BMI.

Incident cases of SNBD were calculated as new positive cases at 12 months of follow-up, amongst cases that were negative at baseline. Prediction analysis was based on logistic regression with SNBD at 12 months follow-up as a dependent binary variable, and dyslipidemia at baseline as an independent binary variable. The same confounding factors were adjusted for. For each model odds ratio (OR), 95% confidence interval (*CI*), and *P*-value were reported. The statistical significance level was set at 0.05.

3. Results

- **Characteristics of the study population**

Among the 2,216 included cases, 1,045 (47%) were females, and 1,171 (53%) were males. The median age was 73 (IQR: 68, 78), and 786 (%) were HC, 1,060 (%) had MCI, and 370 (%) were diagnosed with dementia. The difference in median age between groups was statistically significant (72, 73, and 75 years, respectively, *P*-value <0.001).

At baseline, 357 (16%) study participants had sleep and nighttime behavior disorders, according to their study partner. At 12 months of follow-up, 327 (18%) participants had positive sleep disorder scores, among which 187 were incident cases (fig. 1B).

The median $ADAS_{13}$ total score was 14 (IQR: 9, 22), the median GDS total score was 1.0 (IQR: 0, 2.0), and the median FAQ was 1.0 (IQR: 0, 5.0). Further characteristics of the included cases are presented in Table 1.

- **Comparison between healthy controls, and those with MCI and dementia**

Cholesterol levels ranged from 74 to 476 mg/dL, the median in the total population was 191 mg/dL (IQR: 165, 220), 189 mg/dL (IQR: 165, 217) in HC, 191 mg/dL (IQR: 165, 221) in the MCI group, and 193 mg/dL (IQR: 167, 224) in those with dementia. No statistically significant difference was found between diagnosis groups (*P*-value=0.4). The median Triglyceride level was 120 mg/dL (IQR: 87, 171) in the main population, and ranges between 32 and 2084 mg/dL. No statistically significant difference was found between diagnostic groups (116, 121, and 124 mg/dL, respectively, *P*-value=0.078). The median BMI of the main population was 26.3 (IQR: 23.9, 29.3), ranging between 17.14 and 51.75, with 19 (0.86%) classified as underweight, 796 (35.92%) as healthy weight, 930 (41.97%) as overweight, and 471 (21.25%) as obese. There was a statistically significant difference between BMI medians observed in different groups (26.7 in HC, 26.3 in MCI, and 25.4 in the dementia group, *P*-value <0.001).

- **Comparison between study participants with and without dyslipidemia**

Based on clinical cutoff values, hypercholesterolemia was diagnosed in 920 cases (41.52%), and hypertriglyceridemia in 725 cases (32.72%). Details on the differences between groups are presented in Table 2. BMI was significantly higher in the group without hypercholesterolemia than in those with (26.7 vs. 25.5, *P*-value <0.001). In contrast, BMI was higher in those with hypertriglyceridemia than in those without (25.7 vs. 27.5, *P*-value <0.001). Very weak correlations were found between hypercholesterolemia and hypertriglyceridemia with neurocognitive scores (fig. 2A). BMI showed a decreasing tendency with age, and cases with obesity were significantly younger than those with overweight and

those with a healthy weight (fig. 2B). Furthermore, BMI was negatively correlated with serum Cholesterol levels, and positively correlated with serum Triglyceride levels (fig 2C and 2D). There was no significant difference in Cholesterol levels between those with SNBD at baseline and those without (16% vs. 16%, *P*-value=0.7). Similar results were also observed at 12 months (17% vs. 18%, *P*-value=0.7). Thus, there were more cases of SNBD in the hypertriglyceridemia group than in those with normal triglyceride (19% vs. 14%, *P*-value=0.003). Similarly, 21% of cases of SNBD at 12 months had hypertriglyceridemia and 16% had normal triglyceride levels at baseline (*P*-value=0.025). Cases with SNBD had higher serum Cholesterol levels than those without SNBD but the difference was not statistically significant (193 vs. 191 mg/dL, *P*-value=0.51) (fig. 3A). In contrast, cases with SNBD had significantly higher serum Triglyceride levels than those without (132 vs. 118 mg/dL, *P*-value <0.001) (fig. 3B).

- **Association between sleep and nighttime behavior and dyslipidemia**

At baseline, participants with hypercholesterolemia had 4% higher odds of SNBD but the results were not statistically significant (OR= 1.04, 95% *CI*: 0.83, 1.31, *P*-value=0.744). No statistical significance was observed after adjustment for confounding factors.

Those with hypertriglyceridemia levels had 43% higher odds of SNBD at baseline (OR= 1.43, 95% *CI*: 1.13, 1.80, *P*-value=0.003). The results remained statistically significant after adjustment for confounding factors (adj. OR=1.36, 95% *CI*: 1.06, 1.74, *P*-value=0.016) and after adding BMI to the adjusted model (BMI-adj. OR= 1.29, 95% *CI*: 1.00, 1.66, *P*-value=0.048). BMI and particularly obesity were significantly associated with higher odds of SNBD at baseline. No significant associations were found between hypercholesterolemia and hypertriglyceridemia with incident SNBD at 12 months of follow-up. Detailed results of the logistic regression were reported in Table 3.

4. Discussion

This study explored the association between dyslipidemia based on pathological Cholesterol or Triglyceride levels at baseline, and SNBD reported by the study partner of included older participants at two time points.

The main outcome was the significant association between hypertriglyceridemia and sleep disorders in the cross-sectional analysis at baseline, even after adjusting for age, sex, racial profile, educational level, GDS total score, APOE ε4, cognition-related main diagnosis, and BMI.

- **Sleep duration and dyslipidemia**

Although insomnia is a well-recognized risk factor for cardiovascular and metabolic complications, (24, 25) the significant association between dyslipidemia and sleep disorders extends beyond sleep deprivation. Several population-based studies described a U-shaped association between sleep duration and serum lipid levels, where both short and long sleep durations were significantly associated with dyslipidemia. In people with longer sleep durations, high Triglyceride levels were commonly described. (26-28)

The novelty in the current study was the analysis of associations based on clinical cutoff values defining hypercholesterolemia or hypertriglyceridemia, and a binary outcome defining the existence or not of SNBD. Information on sleep hours was not part of the ADNI investigations, and the focus was SNBD as an outcome rather than sleep duration as an exposure.

- **Sleep quality and dyslipidemia**

In addition to the metabolic effect associated with the quantitative dimension of sleep, lipid levels might also be modulated by the subjective sleep quality. (14) Difficulty in maintaining sleep and excessive daytime sleepiness increased the odds of metabolic syndrome in the

elderly, independently of obesity and snoring. (29) Moreover, the consumption of sleep medication, a biomarker of sleep disorder severity, showed a significant association with elevated low-density lipoprotein-cholesterol (LDL-C). (30) Neither LDL-C nor heigh-density lipoprotein-C (HDL-C) were reported in the main ADNI laboratory data.

- **Sleep dysregulation and dyslipidemia**

Night work, sleep debt, and social jetlag present further risk factors impairing sleep homeostasis and are associated with dyslipidemia and cardiovascular risks. (31, 32) Amongst 5,813 study participants from the Korean National Health and Nutrition Examination Survey (2013-2016), males exercising night work had 53% higher odds of being diagnosed with dyslipidemia. Compared to day-working male participants, male night workers who slept less than six hours and those who skipped meals had significantly higher odds of dyslipidemia. (33)

- **Sleep apnea syndrome and dyslipidemia**

The associations between OSA and metabolic syndrome, (34, 35) as well as the association between OSA and triglyceride-glucose index, a biomarker of insulin resistance, (36) are well-described in the literature. Moreover, novel lipid indices, mainly lipid accumulation product (LAP), visceral adiposity index (VAI), and atherogenic index of plasma (AIP) were found to be higher in people with OSA than controls. (37) Thus, independent of the OSA diagnosis, frequent snoring was also associated with dyslipidemia and predicted linearly higher levels of Triglyceride in a large population study. (38)

- **Associations in younger patients**

The focus of the current study was the elderly with and without cognitive decline. But, the sleep-lipid association was described in younger age groups as well. Higher Triglyceride levels were also reported in adolescents with longer sleep hours, (39) and accelerometry-based

sleep clustering also showed that male adolescents with sleep irregularities had significantly higher Triglyceride levels. (40)

- **Longitudinal studies**

The second outcome was the absence of a significant association between dyslipidemia at baseline and incidental SNBD over a 12-month follow-up period.

While cross-sectional design is inadequate in inferring the causal relationship between sleep and dyslipidemia, longitudinal studies in older adults showed a bidirectional association between sleep duration and blood lipids. Total Cholesterol, LDL-C, HDL-C, and Triglycerides showed different temporal relationships with sleep duration. BMI and age were significant effect modifiers in this association. (41) In a longitudinal population-based cohort of healthy adults, short sleep duration increased the risk of metabolic syndrome, particularly hypertriglyceridemia by 9%. In comparison, long sleep duration decreased the risk of hypertriglyceridemia by 11%. (42)

- **Mechanisms**

The significant association between sleep disorders and higher Triglyceride levels might be explained by concomitant stress, frustration, and consequently eating irregularities. Stress and anxiety are associated with both sleep and eating disorders. (43) First, the association between sleep and stress is bidirectional; an increased emotional stress level might lead to sleep irregularities or insomnia, and sleep disorders might cause higher stress and frustration. (44) Sleep is crucial for emotion regulation and vice versa. (45, 46) Second, sleep disturbance interacts with hormone secretion and eating disorders. (47) Studies have shown that after sleep deprivation, people express eating and appetite dysregulation. (48, 49) Ghrelin, Leptin, and Adiponectin secretion patterns present a mediating effect on the association between sleep duration, and metabolic syndrome. (50, 51) Further, autoimmunity and

neuroinflammation are relevant mechanisms involved in the homeostatic dysregulation associated with sleep disorders, and higher inflammatory biomarkers might further impair metabolic function and energy regulation. (52) Finally, a genetic predisposition, particularly Apolipoprotein genes, might infer the association between sleep and dyslipidemia. (53) The sleep-lipid association was controversially discussed. Linear models seem insufficient to explain the association. (40) Moreover, published results were mainly different depending on the adjustment model used in the analysis. Noteworthy, adjusting for BMI and OSA led to the loss of the statistical significance of the association between sleep duration and quality on one side, and serum Triglyceride and hepatic Triglyceride content on another side. (16) It is known that BMI and OSA infer sleep quality and lipid levels and therefore might present a confounding effect on the path between sleep and dyslipidemia. This contradicts current results since associations remained statistically significant even after adjusting for BMI, and the questions on which the analysis of SNBD was based have mainly a behavioral aspect without considering respiratory symptoms.

- **Strengths**

The major strength of the study is the large number of included cases with high-quality and complete data. The study's inclusion criteria were very restrictive, and only complete cases were considered for the analysis, lowering the bias risk and giving the data a strong analytical value. Older adults tend to be less represented in epidemiological and molecular studies. Therefore, restricting the included population to advanced age groups is a further strength. Older adults have a particularly higher risk of multimorbidity and polymedication. Reducing the risk of one factor might improve the prognosis of other health conditions. This helps lower health costs, in addition to reducing morbidity and mortality risks. Furthermore, the

combination of a cross-sectional and longitudinal design in the analyses allowed a better evaluation of the association's predictive value.

This is the first study exploring the association between informant-reported SNBD and dyslipidemia. Most of the published data studied either OSA or self-reported sleep disorders. In older populations, the self-awareness of sleep quality might be impaired, and this study presents a further strength related to the partner-provided information on sleep-related behavior disorders.

A further strength is related to the fact that the study was based on results obtained from fasting blood. However, a study on the association between sleep disturbances and Triglyceride levels in adolescents showed that the results were not affected by the fasting status of study participants and were statistically significant before and after stratifying by fasting during blood sampling. (40)

- **Limitations**

Despite the interesting findings reported in this study, it is important to acknowledge some limitations.

The first limitation is related to the absence of information on LDL-C and HDL-C levels; both of which are relevant biomarkers of metabolic syndrome and dyslipidemia; but were not assessed in the main laboratory ADNI data. A further limitation is related to the lack of information on comorbid non-alcoholic fatty liver disease (NAFLD) or sarcopenia. Both of them are associated with sleep disorders. (54, 55) In this study, BMI was considered a solid surrogate biomarker for NAFLD, (56, 57) nutrition, and physical activity and was introduced to the adjusted models. Although BMI predicted independently sleep disorders in the univariable models, incorporating BMI in the adjusted models (Model 3) of Cholesterol and

then of Triglyceride did not impact the overall statistical significance of the results. Noteworthy, the association between hypertriglyceridemia and SNBD was not influenced by BMI as covariable and remained statistically significant.

The second limitation is the absence of subjective data such as sleep duration or detailed polysomnographic measurements (Gold Standard). Furthermore, neither information on the objective description of the sleep quality reported by study participants, nor the presence or absence of obstructive sleep apnea were included. While those explorations are of high interest and might have enriched diagnostic methods and information used in the current study, they were largely explored in published data. The informant-based evaluation was favored, particularly because of its relevance in the elderly with cognitive impairment who might lack to some extent self-awareness and tend to over- or underestimate their sleep duration. The questionnaire was oriented toward sleep behavior disorders rather than breathing disorders and associated disturbances.

The third limitation is the lack of information on whether study participants were under lipid-regulating medications. Although this information could be relevant for evaluating the overall prevalence of dyslipidemia in the included sample, the main study objective was to investigate the association between pathological lipid levels at baseline as predicting variable and concomitant sleep disorders, independently of the potential medication effect.

Finally, the study is of predictive value and does not allow drawing causal inferences from the association between dyslipidemia and SNBD.

4. Conclusions

Hypertriglyceridemia, but not hypercholesterolemia, was associated with SNBD in older adults at baseline. This association was independent of BMI. However, none of the dyslipidemia

forms did predict incidental cases of SNBD over a follow-up period of 12 months. The current study emphasizes the importance of systematic screening of sleep disorders in older patients and its adapted management in mitigating metabolic risks and preventing related cardiovascular complications. Informant-based interviews are helpful and provide complementary information on sleep disorders in older individuals with cognitive impairment.

Declarations

Ethics approval and consent to participate: Ethical approvals were obtained from local IRBs corresponding to every recruitment center of ADNI. Participants gave written consent. Information can be found at https://adni.loni.usc.edu.

Consent for publication: Not applicable.

Availability of data and materials: Data can be found at https://adni.loni.usc.edu.

Competing interest: None.

Declaration of funding: AH did not receive any specific grant from funding agencies in the public, commercial, or not-for-profit sector. Data collection and sharing for ADNI project was funded by the Alzheimer's Disease Neuroimaging Initiative (ADNI; National Institutes of Health Grant U19 AG024904). ADNI is made possible with funding from the NIH and private sector support detailed at https://adni.loni.usc.edu/about/ and in the Acknowledgment part.

Author contributions: AH has full access to all of the data and takes responsibility for the integrity of the data and the accuracy of the analysis, visualization, drafting, and editing of the manuscript.

*Data used in preparation of this article were obtained from the Alzheimer's Disease Neuroimaging Initiative (ADNI) database (adni.loni.usc.edu). As such, the investigators within

the ADNI contributed to the design and implementation of ADNI and/or provided data but did not participate in analysis or writing of this report. A complete listing of ADNI investigators can be found at: http://adni.loni.usc.edu/wp-content/uploads/how_to_apply/ADNI_Acknowledgement_List.pdf

Acknowledgments: "Data collection and sharing for the Alzheimer's Disease Neuroimaging Initiative (ADNI) is funded by the National Institute on Aging (National Institutes of Health Grant U19 AG024904). The grantee organization is the Northern California Institute for Research and Education. In the past, ADNI has also received funding from the National Institute of Biomedical Imaging and Bioengineering, the Canadian Institutes of Health Research, and private sector contributions through the Foundation for the National Institutes of Health (FNIH) including generous contributions from the following: AbbVie, Alzheimer's Association; Alzheimer's Drug Discovery Foundation; Araclon Biotech; BioClinica, Inc.; Biogen; Bristol-Myers Squibb Company; CereSpir, Inc.; Cogstate; Eisai Inc.; Elan Pharmaceuticals, Inc.; Eli Lilly and Company; EuroImmun; F. Hoffmann-La Roche Ltd and its affiliated company Genentech, Inc.; Fujirebio; GE Healthcare; IXICO Ltd.; Janssen Alzheimer Immunotherapy Research & Development, LLC.; Johnson & Johnson Pharmaceutical Research &Development LLC.; Lumosity; Lundbeck; Merck & Co., Inc.; Meso Scale Diagnostics, LLC.; NeuroRx Research; Neurotrack Technologies; Novartis Pharmaceuticals Corporation; Pfizer Inc.; Piramal Imaging; Servier; Takeda Pharmaceutical Company; and Transition Therapeutics."

References

1. Sateia MJ. International Classification of Sleep Disorders-Third Edition. Chest. 2014;146(5):1387-94.

2. The Lancet D, amp, Endocrinology. Sleep: a neglected public health issue. The Lancet Diabetes & Endocrinology. 2024;12(6):365.

3. Chen B, Vgontzas AN, Li Y. Good sleep and health. The Lancet Healthy Longevity. 2023;4(7):e295-e6.

4. Siddiquee AT, Lee SK, Kim S, Lee MH, Kim HJ, Shin C. All-cause and major-cause mortality associated with sleep latency in the Korean Genome and Epidemiology Study (KoGES): a population-based prospective cohort study. Lancet Healthy Longev. 2023;4(7):e316-e25.

5. Benjafield AV, Ayas NT, Eastwood PR, Heinzer R, Ip MSM, Morrell MJ, et al. Estimation of the global prevalence and burden of obstructive sleep apnoea: a literature-based analysis. The Lancet Respiratory Medicine. 2019;7(8):687-98.

6. Spinedi E, Cardinali DP. Neuroendocrine-Metabolic Dysfunction and Sleep Disturbances in Neurodegenerative Disorders: Focus on Alzheimer's Disease and Melatonin. Neuroendocrinology. 2019;108(4):354-64.

7. Hung CM, Li YC, Chen HJ, Lu K, Liang CL, Liliang PC, et al. Risk of dementia in patients with primary insomnia: a nationwide population-based case-control study. BMC Psychiatry. 2018;18(1):38.

8. Yaffe K, Falvey CM, Hoang T. Connections between sleep and cognition in older adults. The Lancet Neurology. 2014;13(10):1017-28.

9. Sabia S, Fayosse A, Dumurgier J, van Hees VT, Paquet C, Sommerlad A, et al. Association of sleep duration in middle and old age with incidence of dementia. Nature Communications. 2021;12(1):2289.

10. Oh JY, Walsh CM, Ranasinghe K, Mladinov M, Pereira FL, Petersen C, et al. Subcortical Neuronal Correlates of Sleep in Neurodegenerative Diseases. JAMA Neurology. 2022;79(5):498-508.

11. Lim DC, Najafi A, Afifi L, Bassetti CLA, Buysse DJ, Han F, et al. The need to promote sleep health in public health agendas across the globe. The Lancet Public Health. 2023;8(10):e820-e6.

12. Zhang S, Xie L, Yu H, Zhang W, Qian B. Association between nighttime-daytime sleep patterns and chronic diseases in Chinese elderly population: a community-based cross-sectional study. BMC Geriatr. 2019;19(1):124.

13. Alberti KGMM, Zimmet P, Shaw J. The metabolic syndrome—a new worldwide definition. The Lancet. 2005;366(9491):1059-62.

14. Okubo N, Matsuzaka M, Takahashi I, Sawada K, Sato S, Akimoto N, et al. Relationship between self-reported sleep quality and metabolic syndrome in general population. BMC Public Health. 2014;14:562.

15. Wang D, Chen J, Zhou Y, Ma J, Zhou M, Xiao L, et al. Association between sleep duration, sleep quality and hyperlipidemia in middle-aged and older Chinese: The Dongfeng-Tongji Cohort Study. Eur J Prev Cardiol. 2019;26(12):1288-97.

16. Bos MM, Noordam R, van den Berg R, de Mutsert R, Rosendaal FR, Blauw GJ, et al. Associations of sleep duration and quality with serum and hepatic lipids: The Netherlands Epidemiology of Obesity Study. J Sleep Res. 2019;28(4):e12776.

17. Zheng Y, Wang A, Pan C, Lu J, Dou J, Lu Z, et al. Impact of night sleep duration on glycemic and triglyceride levels in Chinese with different glycemic status. J Diabetes. 2015;7(1):24-30.

18. von Elm E, Altman DG, Egger M, Pocock SJ, Gøtzsche PC, Vandenbroucke JP. The Strengthening the Reporting of Observational Studies in Epidemiology (STROBE) statement: guidelines for reporting observational studies. The Lancet. 2007;370(9596):1453-7.

19. Report of the National Cholesterol Education Program Expert Panel on Detection, Evaluation, and Treatment of High Blood Cholesterol in Adults. The Expert Panel. Arch Intern Med. 1988;148(1):36-69.

20. Third Report of the National Cholesterol Education Program (NCEP) Expert Panel on Detection, Evaluation, and Treatment of High Blood Cholesterol in Adults (Adult Treatment Panel III) Final Report. Circulation. 2002;106(25):3143-.

21. Cummings JL, Mega M, Gray K, Rosenberg-Thompson S, Carusi DA, Gornbein J. The Neuropsychiatric Inventory. Neurology. 1994;44(12):2308-.

22. Kaufer DI, Cummings JL, Ketchel P, Smith V, MacMillan A, Shelley T, et al. Validation of the NPI-Q, a Brief Clinical Form of the Neuropsychiatric Inventory. The Journal of Neuropsychiatry and Clinical Neurosciences. 2000;12(2):233-9.

23. Garrow JS, Webster J. Quetelet's index (W/H2) as a measure of fatness. Int J Obes. 1985;9(2):147-53.

24. Lee MS, Shin JS, Lee J, Lee YJ, Kim MR, Park KB, et al. The association between mental health, chronic disease and sleep duration in Koreans: a cross-sectional study. BMC Public Health. 2015;15:1200.

25. Jin B, Zhang H, Song F, Wu G, Yang H. Interaction of sleep duration and depression on cardiovascular disease: a retrospective cohort study. BMC Public Health. 2022;22(1):1752.

26. Liu X, Huang L, Wu Q, Chen Y, Chen X, Chen H, et al. Sleep characteristic profiles and the correlation with spectrum of metabolic syndrome among older adult: a cross-sectional study. BMC Geriatr. 2022;22(1):414.

27. Du J, Chen Y, Zhou N, Song Y, Wang W, Hong X. Associations between self-reported sleep duration and abnormal serum lipids in eastern China: a population-based cross-sectional survey. Sleep Med. 2022;95:1-8.

28. Full KM, Gallo LC, Malhotra A, Bellettiere J, Kerr J, Arredondo E, et al. Modeling the cardiometabolic benefits of sleep in older women: exploring the 24-hour day. Sleep. 2020;43(1).

29. Akbaraly TN, Jaussent I, Besset A, Bertrand M, Barberger-Gateau P, Ritchie K, et al. Sleep complaints and metabolic syndrome in an elderly population: the Three-City Study. Am J Geriatr Psychiatry. 2015;23(8):818-28.

30. Vozoris NT. Insomnia Symptoms Are Not Associated with Dyslipidemia: A Population-Based Study. Sleep. 2016;39(3):551-8.

31. Cabeza de Baca T, Chayama KL, Redline S, Slopen N, Matsushita F, Prather AA, et al. Sleep debt: the impact of weekday sleep deprivation on cardiovascular health in older women. Sleep. 2019;42(10).

32. Sládek M, Klusáček J, Hamplová D, Sumová A. Population-representative study reveals cardiovascular and metabolic disease biomarkers associated with misaligned sleep schedules. Sleep. 2023;46(6).

33. Joo JH, Lee DW, Choi DW, Park EC. Association between night work and dyslipidemia in South Korean men and women: a cross-sectional study. Lipids Health Dis. 2019;18(1):75.

34. Geovanini GR, Wang R, Weng J, Jenny NS, Shea S, Allison M, et al. Association between Obstructive Sleep Apnea and Cardiovascular Risk Factors: Variation by Age, Sex, and Race. The Multi-Ethnic Study of Atherosclerosis. Ann Am Thorac Soc. 2018;15(8):970-7.

35. Toyama Y, Chin K, Chihara Y, Takegami M, Takahashi KI, Sumi K, et al. Association between sleep apnea, sleep duration, and serum lipid profile in an urban, male, working population in Japan. Chest. 2013;143(3):720-8.

36. Behnoush AH, Khalaji A, Ghondaghsaz E, Masrour M, Shokri Varniab Z, Khalaji S, et al. Triglyceride-glucose index and obstructive sleep apnea: a systematic review and meta-analysis. Lipids in Health and Disease. 2024;23(1):4.

37. Behnoush AH, Bahiraie P, Shokri Varniab Z, Foroutani L, Khalaji A. Composite lipid indices in patients with obstructive sleep apnea: a systematic review and meta-analysis. Lipids Health Dis. 2023;22(1):84.

38. Tian Y, Li D, Mu H, Wei S, Guo D. Positive correlation between snoring and dyslipidemia in adults: results from NHANES. Lipids Health Dis. 2023;22(1):73.

39. Anni NS, Jung SJ, Shim JS, Jeon YW, Lee GB, Kim HC. Stressful life events and serum triglyceride levels: the Cardiovascular and Metabolic Diseases Etiology Research Center cohort in Korea. Epidemiol Health. 2021;43:e2021042.

40. Wang M, Flexeder C, Harris CP, Thiering E, Koletzko S, Bauer CP, et al. Accelerometry-assessed sleep clusters and cardiometabolic risk factors in adolescents. Obesity (Silver Spring). 2024;32(1):200-13.

41. Chen Z, Zhang X, Duan Y, Mo T, Liu W, Ma Y, et al. The Relationship Between Sleep Duration and Blood Lipids Among Chinese Middle-Aged and Older Adults: Cross-Lagged Path Analysis From CHARLS. Front Public Health. 2022;10:868059.

42. Deng HB, Tam T, Zee BC, Chung RY, Su X, Jin L, et al. Short Sleep Duration Increases Metabolic Impact in Healthy Adults: A Population-Based Cohort Study. Sleep. 2017;40(10).

43. Ong SH, Wickramaratne P, Tang M, Weissman MM. Early childhood sleep and eating problems as predictors of adolescent and adult mood and anxiety disorders. J Affect Disord. 2006;96(1-2):1-8.

44. Balter LJT, Sundelin T, Axelsson J. Sickness and sleep health predict frustration and affective responses to a frustrating trigger. Sci Rep. 2021;11(1):1542.

45. Palmer CA, Alfano CA. Sleep and emotion regulation: An organizing, integrative review. Sleep Medicine Reviews. 2017;31:6-16.

46. Demichelis OP, Grainger SA, Burr L, Henry JD. Emotion regulation mediates the effects of sleep on stress and aggression. Journal of Sleep Research. 2023;32(3):e13787.

47. Mehr JB, Mitchison D, Bowrey HE, James MH. Sleep dysregulation in binge eating disorder and “food addiction”: the orexin (hypocretin) system as a potential neurobiological link. Neuropsychopharmacology. 2021;46(12):2051-61.

48. Barragán R, Zuraikat FM, Tam V, RoyChoudhury A, St-Onge MP. Changes in eating patterns in response to chronic insufficient sleep and their associations with diet quality: a randomized trial. J Clin Sleep Med. 2023;19(11):1867-75.

49. Liu S, Wang X, Zheng Q, Gao L, Sun Q. Sleep Deprivation and Central Appetite Regulation. Nutrients. 2022;14(24).

50. Li L, Fu J, Yu XT, Li G, Xu L, Yin J, et al. Sleep Duration and Cardiometabolic Risk Among Chinese School-aged Children: Do Adipokines Play a Mediating Role? Sleep. 2017;40(5).

51. Chaput JP, McHill AW, Cox RC, Broussard JL, Dutil C, da Costa BGG, et al. The role of insufficient sleep and circadian misalignment in obesity. Nat Rev Endocrinol. 2023;19(2):82-97.

52. Nowakowski S, Matthews KA, von Känel R, Hall MH, Thurston RC. Sleep characteristics and inflammatory biomarkers among midlife women. Sleep. 2018;41(5).

53. Yang L, Ma L, Guo W, Fang Q, Lai X, Zhang X. Interaction of polymorphisms in APOA4-APOA5-ZPR1-BUD13 gene cluster and sleep duration on 5-year lipid changes in middle aged and older Chinese. Sleep. 2019;42(8).

54. Wei Y-T, Lee P-Y, Lin C-Y, Chen H-J, Lin C-C, Wu J-S, et al. Non-alcoholic fatty liver disease among patients with sleep disorders: a Nationwide study of Taiwan. BMC Gastroenterology. 2020;20(1):32.

55. Liu K, Luo J, Chen Y, Li B, Tian Y, Wang X, et al. Association between sarcopenia and sleep disorders: a cross-sectional population based study. Front Nutr. 2024;11:1415743.

56. Fan R, Wang J, Du J. Association between body mass index and fatty liver risk: A dose-response analysis. Scientific Reports. 2018;8(1):15273.

57. Wang M, Chang M, Shen P, Wei W, Li H, Shen G. Application value of triglyceride-glucose index and triglyceride-glucose body mass index in evaluating the degree of hepatic steatosis in non-alcoholic fatty liver disease. Lipids in Health and Disease. 2023;22(1):186.

<u>Captions</u>

<u>Tables</u>

Table 1: Characteristics of the study population and comparison between healthy controls, and those with MCI and dementia

Table 2: Comparison between study participants with and without dyslipidemia

Table 3: Association between sleep and nighttime behavior and dyslipidemia at baseline and 12 months follow-up

<u>Figures</u>

Figure 1: Characteristics of the study population

Figure 1 A: Chart flow of included studies

Figure 1 B: Classification of cases during study follow-up

Figure 2: BMI categories and correlation analyses

Figure 2 A: Spearman correlation plot

Figure 2 B: Median age across different BMI categories

Figure 2 C: Spearman correlation between Cholesterol and BMI

Figure 2 D: Spearman correlation between Triglyceride and BMI

Figure 3: Dyslipidemia and sleep disorders

Figure 3 A: Cholesterol levels and sleep disorders

Figure 3 B: Triglyceride levels and sleep disorders

Table 1: Characteristics of the study population and comparison between healthy controls, and those with MCI and dementia

Characteristic	*N*	*Overall,* *N = 2,216*[1]	*Healthy controls* *N = 786*[1]	*MCI* *N = 1,060*[1]	*Dementia* *N = 370*[1]	*P-value*[2]
Age (years)	2,216	73 (68, 78)	72 (68, 77)	73 (68, 78)	75 (70, 80)	**<0.001**
Sex	2,216					**<0.001**
Female		1,045 (47%)	440 (56%)	442 (42%)	163 (44%)	
Male		1,171 (53%)	346 (44%)	618 (58%)	207 (56%)	
Educational level (years)	2,216	16.00 (14.00, 18.00)	16.00 (15.00, 18.00)	16.00 (14.00, 18.00)	16.00 (13.00, 18.00)	**<0.001**
Marital status	2,216					**<0.001**
Currently married		1,673 (75%)	547 (70%)	815 (77%)	311 (84%)	
Currently not married or unknown		543 (25%)	239 (30%)	245 (23%)	59 (16%)	
Home	2,216					0.8
House or appartment		2,101 (95%)	748 (95%)	1,006 (95%)	347 (94%)	
Retirement or nursing institution		76 (3.4%)	26 (3.3%)	34 (3.2%)	16 (4.3%)	
Other		39 (1.8%)	12 (1.5%)	20 (1.9%)	7 (1.9%)	
Racial profile	2,216					**<0.001**
White		1,952 (88%)	651 (83%)	963 (91%)	338 (91%)	
Black		167 (7.5%)	92 (12%)	56 (5.3%)	19 (5.1%)	
Other		97 (4.4%)	43 (5.5%)	41 (3.9%)	13 (3.5%)	
APOE ε4 status	2,031					**<0.001**
0 allele		1,095 (54%)	488 (70%)	495 (50%)	112 (32%)	
1 allele		735 (36%)	188 (27%)	378 (39%)	169 (48%)	
2 alleles		201 (9.9%)	22 (3.2%)	108 (11%)	71 (20%)	
Missing values		185	88	79	18	
$ADAS_{13}$ total score	2,216	14 (9, 22)	8 (5, 12)	16 (11, 21)	29 (24, 34)	**<0.001**
MMSE total score	2,216	28.00 (26.00, 29.00)	29.00 (29.00, 30.00)	28.00 (26.00, 29.00)	23.00 (21.00, 25.00)	**<0.001**
CDR-SB	2,216	1.00 (0.00, 2.00)	0.00 (0.00, 0.00)	1.50 (1.00, 2.00)	4.50 (3.50, 5.00)	**<0.001**
FAQ total score	2,211	1.0 (0.0, 5.0)	0.0 (0.0, 0.0)	1.0 (0.0, 5.0)	13.0 (8.0, 18.0)	**<0.001**
Missing values		5	0	4	1	
GDS total score	2,216	1.00 (0.00, 2.00)	0.00 (0.00, 1.00)	1.00 (1.00, 3.00)	1.00 (1.00, 3.00)	**<0.001**
BMI	2,216	26.3 (23.9, 29.3)	26.7 (24.1, 30.0)	26.3 (24.0, 29.2)	25.4 (23.1, 28.0)	**<0.001**
Cholesterol levels (mg/dL)	2,216	191 (165, 220)	189 (165, 217)	191 (165, 221)	193 (167, 224)	0.4
Triglyceride levels (mg/dL)	2,216	120 (87, 171)	116 (85, 164)	121 (86, 177)	124 (93, 171)	0.078
Sleep disorders at baseline	2,216	357 (16%)	80 (10%)	193 (18%)	84 (23%)	**<0.001**
Sleep disorders at 12 months	1,845	327 (18%)	56 (8.7%)	200 (22%)	71 (23%)	**<0.001**
Missing values		371	141	163	67	

[1] *Median (IQR); n (%),* [2] *Kruskal-Wallis rank sum test; Pearson's Chi-squared test*

$ADAS_{13}$: *Alzheimer's Disease Assessment Score – 13 items -* ***APOE ε4***: *Apolipoprotein E ε4 –* ***BMI***: *Body-Mass Index (Weight in Kg / (Height in m)^2) -* ***CDR- SB***: *Clinical Dementia Rating Scale - sum of boxes -* ***FAQ***: *Functional Activities Questionnaire -* ***GDS***: *Geriatric Depression Scale –* ***MCI***: *Mild Cognitive Impairment -* ***MMSE***: *Mini-Mental Status Examination*

Table 2: Comparison between study participants with and without dyslipidemia

Characteristic	** *Hypercholesterolemia* **			** *Hypertriglyceridemia* **		
	**Normal (<200 mg/dL)** N = 1,296[1]	**High (≥200 mg/dL)** N = 920[1]	***P*-value**[2]	**Normal (<150 mg/dL)** N = 1,491[1]	**High (≥150 mg/dL)** N = 725[1]	***P*-value**[2]
Age (years)	74 (69, 79)	72 (67, 78)	**<0.001**	73 (68, 78)	73 (67, 78)	0.3
Sex			**<0.001**			0.4
Female	453 (35%)	592 (64%)		713 (48%)	332 (46%)	
Male	843 (65%)	328 (36%)		778 (52%)	393 (54%)	
Main cognitive diagnosis			0.6			0.3
Healthy controls	465 (36%)	321 (35%)		543 (36%)	243 (34%)	
MCI	623 (48%)	437 (48%)		698 (47%)	362 (50%)	
Dementia	208 (16%)	162 (18%)		250 (17%)	120 (17%)	
Educational level (years)	16.00 (14.00, 18.00)	16.00 (14.00, 18.00)	0.4	16.00 (14.00, 18.00)	16.00 (14.00, 18.00)	**<0.001**
Marital status			**0.004**			0.6
Currently married	1,007 (78%)	666 (72%)		1,131 (76%)	542 (75%)	
Currently not married or unknown	289 (22%)	254 (28%)		360 (24%)	183 (25%)	
Home			0.4			0.5
House or appartment	1,230 (95%)	871 (95%)		1,419 (95%)	682 (94%)	
Retirement or nursing institution	47 (3.6%)	29 (3.2%)		47 (3.2%)	29 (4.0%)	
Other	19 (1.5%)	20 (2.2%)		25 (1.7%)	14 (1.9%)	
Racial profile			0.6			**<0.001**
White	1,140 (88%)	812 (88%)		1,294 (87%)	658 (91%)	
Black	95 (7.3%)	72 (7.8%)		137 (9.2%)	30 (4.1%)	
Other	61 (4.7%)	36 (3.9%)		60 (4.0%)	37 (5.1%)	
APOE ε4 status			**0.024**			0.3
0 allele	663 (56%)	432 (51%)		727 (53%)	368 (55%)	
1 allele	411 (35%)	324 (38%)		489 (36%)	246 (37%)	
2 alleles	104 (8.8%)	97 (11%)		145 (11%)	56 (8.4%)	
Missing values	118	67		130	55	
ADAS$_{13}$ total score	14 (9, 21)	14 (8, 22)	0.059	14 (9, 21)	14 (9, 22)	0.4
MMSE total score	28.00 (26.00, 29.00)	28.00 (26.00, 30.00)	>0.9	28.00 (26.00, 29.00)	28.00 (26.00, 29.00)	0.2
CDR-SB	1.00 (0.00, 2.00)	1.00 (0.00, 2.50)	0.4	1.00 (0.00, 2.00)	1.00 (0.00, 2.50)	0.081
FAQ total score	1.0 (0.0, 5.0)	1.0 (0.0, 5.0)	0.7	0.0 (0.0, 5.0)	1.0 (0.0, 6.0)	**0.028**
Missing values	1	4		4	1	
GDS total score	1.00 (0.00, 2.00)	1.00 (0.00, 2.00)	0.7	1.00 (0.00, 2.00)	1.00 (0.00, 2.00)	**<0.001**
BMI	26.7 (24.4, 29.8)	25.5 (23.1, 28.7)	**<0.001**	25.7 (23.4, 28.6)	27.5 (25.1, 30.9)	**<0.001**
Cholesterol (mg/dL)	170 (151, 184)	225 (212, 246)	**<0.001**	187 (162, 216)	197 (172, 227)	**<0.001**
Triglyceride (mg/dL)	115 (84, 162)	127 (92, 183)	**<0.001**	98 (76, 120)	203 (172, 255)	**<0.001**

Sleep disorders at baseline	206 (16%)	151 (16%)	0.7	216 (14%)	141 (19%)	**0.003**
Sleep disorders at 12 months	188 (17%)	139 (18%)	0.7	199 (16%)	128 (21%)	**0.025**
Missing values	215	156		270	101	

[1] *Median (IQR); n (%) -* [2] *Wilcoxon rank sum test; Pearson's Chi-squared test*

ADAS$_{13}$: *Alzheimer's Disease Assessment Score – 13 items -* ***APOE ε4***: *Apolipoprotein E ε4 –* ***BMI***: *Body-Mass Index (Weight in Kg / (Height in m)*^{2}*) -* ***CDR- SB***: *Clinical Dementia Rating Scale - sum of boxes -* ***FAQ***: *Functional Activities Questionnaire -* ***GDS***: *Geriatric Depression Scale –* ***MCI***: *Mild Cognitive Impairment -* ***MMSE***: *Mini-Mental Status Examination*

Table 3: Association between sleep and nighttime behavior and dyslipidemia at baseline and 12 months follow-up

Characteristic	*Model 1*				*Model 2*				*Model 3*			
	N	*Event*	*OR[1] (95% CI[1])*	*P-value*	*N*	*Event*	*OR[1] (95% CI[1])*	*P-value*	*N*	*Event*	*OR[1] (95% CI[1])*	*P-value*
					Sleep disorders at baseline							
Cholesterol	2,216	357		0.744	2,031	335		0.412	2,031	335		0.571
Normal (<200 mg/dL)			—				—				—	
High (≥200 mg/dL)			1.04 (0.83, 1.31)				0.90 (0.70, 1.16)				0.93 (0.72, 1.20)	
Triglyceride	2,216	357		**0.003**	2,031	335		**0.016**	2,031	335		**0.048**
Normal (<150 mg/dL)			—				—				—	
High (≥150 mg/dL)			1.43 (1.13, 1.80)				1.36 (1.06, 1.74)				1.29 (1.00, 1.66)	
BMI	2,216	357	1.03 (1.00, 1.05)	**0.030**	2,031	335	1.03 (1.01, 1.06)	**0.008**			—	
BMI categories	2,216	357		0.095	2,031	335		**0.029**			—	
Healthy Weight			—				—				—	
Underweight			1.12 (0.26, 3.43)	0.857			0.95 (0.21, 2.99)	0.934			—	
Overweight			1.12 (0.86, 1.47)	0.391			1.14 (0.86, 1.51)	0.371			—	
Obesity			1.47 (1.09, 1.99)	0.012			1.65 (1.18, 2.29)	**0.003**			—	
					Incident sleep disorders at 12 months follow-up							
Cholesterol	1,550	187		0.719	1,468	178		0.641	1,468	178		0.706
Normal (<200 mg/dL)			—				—				—	
High (≥200 mg/dL)			1.06 (0.77, 1.44)				1.08 (0.77, 1.52)				1.07 (0.76, 1.50)	
Triglyceride	1,550	187		0.066	1,468	178		0.167	1,468	178		0.122
Normal (<150 mg/dL)			—				—				—	
High (≥150 mg/dL)			1.35 (0.98, 1.85)				1.27 (0.90, 1.76)				1.31 (0.93, 1.83)	
BMI	1,550	187	0.98 (0.95, 1.01)	0.202	1,468	178	0.98 (0.95, 1.02)	0.417			—	
BMI categories	1,550	187		**0.046**	1,468	178		0.090			—	
Healthy Weight			—				—				—	
Underweight			4.81 (1.23, 16.4)	**0.014**			4.15 (1.04, 14.6)	**0.030**			—	
Overweight			1.34 (0.95, 1.90)	0.101			1.35 (0.93, 1.95)	0.114			—	
Obesity			0.97 (0.61, 1.51)	0.891			1.02 (0.62, 1.66)	0.941			—	

[1] *OR = Odds Ratio, CI = Confidence Interval, BMI = Body Mass Index*

Model 1*: crude logistic regression analysis,* ***Model 2****: adjusted for age, sex, racial profile, educational level, cognition-related main diagnosis, geriatric depression scale total score, APOE ε4 status,* ***Model 3****: model 2 + BMI*

Sleep and nighttime behavior disorders in older adults: associations with hypercholesterolemia and hypertriglyceridemia at baseline and a prediction analysis of incidental cases at 12 months follow-up

ORIGINALITY REPORT

74%
SIMILARITY INDEX

PRIMARY SOURCES

	Source	Match
1	Asma Hallab. "High serum Cholesterol and Triglyceride levels in older adults: associations with sleep and nighttime behavior disorders at baseline and a prediction analysis of incidental cases at 12 months follow-up", Cold Spring Harbor Laboratory, 2024<br>Crossref Posted Content	5914 words — 66%
2	sciety.org<br>Internet	213 words — 2%
3	Asma Hallab. "Sex-modulated association between thyroid stimulating hormone and informant-perceived anxiety in non-depressed older adults: prediction models and relevant cutoff value", Cold Spring Harbor Laboratory, 2024<br>Crossref Posted Content	117 words — 1%
4	www.frontiersin.org<br>Internet	50 words — 1%
5	cardiab.biomedcentral.com<br>Internet	41 words — < 1%

6 Amir Hossein Behnoush, Asma Mousavi, Elina Ghondaghsaz, Shayan Shojaei, Alessandro Cannavo, Amirmohammad Khalaji. "The importance of assessing the triglyceride-glucose index (TyG) in patients with depression: A systematic review", Neuroscience & Biobehavioral Reviews, 2024
Crossref
35 words — < 1%

7 www.researchsquare.com
Internet
35 words — < 1%

8 lipidworld.biomedcentral.com
Internet
33 words — < 1%

9 Asma Hallab. "Sex-, and neurodegeneration-dependent effect modification in the association between thyroid function and cognitive impairment in non-depressed, non-demented elderly", Cold Spring Harbor Laboratory, 2024
Crossref Posted Content
31 words — < 1%

10 www.biomedicineonline.org
Internet
24 words — < 1%

11 bio-protocol.org
Internet
16 words — < 1%

12 discovery.researcher.life
Internet
16 words — < 1%

13 dergipark.org.tr
Internet
11 words — < 1%

14 pubmed.ncbi.nlm.nih.gov
Internet
10 words — < 1%

15 Bloniecki Kallio, Victor. "Using CSF Biomarkers to Understand Mechanisms of Behavioral Changes and Effects of Drug Treatment in Dementia.", Karolinska Institutet (Sweden), 2021
ProQuest
9 words — < 1%

16 Tesfaye, Rackeb. "The Interplay Between Sleep, Cognition, and Copy Number Variations in Autism Spectrum Disorder.", McGill University (Canada), 2023
ProQuest
9 words — < 1%

17 Shanker Swaminathan. "Amyloid pathway-based candidate gene analysis of [11C]PiB-PET in the Alzheimer's Disease Neuroimaging Initiative (ADNI) cohort", Brain Imaging and Behavior, 09/08/2011
Crossref
8 words — < 1%

18 Tissot, Cécile. "The Impact of Tau Biomarkers in the A/T/(N) Framework of Alzheimer's Disease", McGill University (Canada), 2023
ProQuest
8 words — < 1%

19 ibdigital.uib.es
Internet
8 words — < 1%

20 journals.plos.org
Internet
8 words — < 1%

21 ouci.dntb.gov.ua
Internet
8 words — < 1%

22 pesquisa.bvsalud.org
Internet
8 words — < 1%

23 worldwidescience.org
Internet
8 words — < 1%

24	www.science.gov<br>Internet	8 words — < 1%
25	Sepideh Shokouhi, John W. Mckay, Suzanne L. Baker, Hakmook Kang et al. "Reference tissue normalization in longitudinal 18F-florbetapir positron emission tomography of late mild cognitive impairment", Alzheimer's Research & Therapy, 2016<br>Crossref	6 words — < 1%

EXCLUDE QUOTES	OFF	EXCLUDE SOURCES	OFF
EXCLUDE BIBLIOGRAPHY	OFF	EXCLUDE MATCHES	OFF
